# Practices and Perspectives of Traditional Bone Setters in Northern Tanzania

**DOI:** 10.5334/aogh.2878

**Published:** 2020-06-16

**Authors:** Elizabeth B. Card, Joy E. Obayemi, Octavian Shirima, Matayo Lazaro, Honest Massawe, John W. Stanifer, Ajay Premkumar, Neil P. Sheth

**Affiliations:** 1 Perelman School of Medicine, University of Pennsylvania, Philadelphia, PA, US; 2 Department of Orthopaedic Surgery, Kilimanjaro Christian Medical Center, Moshi, TZ; 3 Kilimanjaro Clinical Research Institute, Moshi, TZ; 4 Munson Nephrology, Munson Medical Center, Traverse City, MI, US; 5 Department of Orthopaedic Surgery, Hospital for Special Surgery, New York, NY, US; 6 Department of Orthopaedic Surgery, University of Pennsylvania, Philadelphia, PA, US

## Abstract

**Background:**

Traditional health practitioners remain a critical source of care in Tanzania, more than 50% of Tanzanians frequently using their services. With a severe shortage of orthopaedic surgeons (1:3.3 million Tanzanians) traditional bone setters (TBSs) could potentially expand access to musculoskeletal care and improve outcomes for morbidity as a result of trauma.

**Objective:**

We sought to identify the advantages and disadvantages of traditional bone setting in Tanzania and to assess potential for collaboration between TBSs and allopathic orthopaedic surgeons.

**Methods:**

Between June and July 2017 we interviewed six TBSs identified as key informants in the regions of Kilimanjaro, Arusha, and Manyara. We conducted semi-structured interviews about practices and perspectives on allopathic healthcare, and analyzed the data using a deductive framework method.

**Findings:**

The TBSs reported that their patients were primarily recruited from their local communities via word-of-mouth communication networks. Payment methods for services included bundling costs, livestock barter, and sliding scale pricing. Potentially unsafe practices included lack of radiographic imaging to confirm reduction; cutting and puncturing of skin with unsterile tools; and rebreaking healed fractures. The TBSs described past experience collaborating with allopathic healthcare providers, referring patients to hospitals, and utilizing allopathic techniques in their practice. All expressed enthusiasm in future collaboration with allopathic hospitals.

**Conclusions:**

TBSs confer the advantages of word-of-mouth communication networks and greater financial and geographic accessibility. However, some of their practices raise concerns relating to infection, fracture malunion or nonunion, and iatrogenic trauma from manipulating previously healed fractures. A formal collaboration between TBSs and orthopaedic surgeons, based on respect and regular communication, could alleviate concerns through the development of care protocols and increase access to optimal orthopaedic care through a standardized triage and follow-up system.

## Introduction

Despite the growth of allopathic healthcare sectors across Africa, traditional medicines and medical practices remain important and primary sources of healthcare for most of the population [1,2,3,4]. In the United Republic of Tanzania, an estimated 60% of the population utilizes traditional medicine to meet their healthcare needs [5]. Traditional medical practices encompass a broad range of specialties, including traditional bone setting for care of musculoskeletal injury. Bone setting is one of the most widely recognized and used forms of traditional medical practice, with an estimated 10–40% of people using it worldwide [6,7].

With the rise in road traffic accidents and the burden of musculoskeletal injury in Sub-Saharan Africa, there has been growing need for musculoskeletal care [8]. Some countries meet this demand through traditional bone setting, especially due to limited access to allopathic orthopaedic care [4,9,10,11]. However, there is concern among the allopathic sector regarding complications that can arise from bone setting such as malunion. Many have tried to understand traditional bone setting practices with the goal of reducing negative outcomes [2,12,13,14,15]. Some studies recommend a complete phasing out of bone setting, while others, recognizing the futility of attempting to uproot an entrenched art patronized by all levels of society, have piloted training interventions to reduce complications from traditional bone setting [14,16,17]. Others call to train and incorporate traditional bone setters (TBSs) into the allopathic health system to take advantage of their strengths such as geographic, financial, and cultural accessibility [13,15].

Tanzania has a severe shortage of orthopaedic surgeons and a large reliance on traditional medicine, but the specific role of TBSs in alleviating the burden of musculoskeletal disease has yet to be clearly defined [18,19,20,21,22,23]. The recent legalization of traditional medicine in Tanzania and past success with traditional practitioner training may welcome collaboration to increase access to orthopaedic care and reduce complications associated with traditional bone setting [5,19,24,25]. Before such interventions can take place, TBS backgrounds, practices, and views towards allopathic healthcare must be elucidated to judge willingness and method for collaboration. Our study is the first to answer questions in these areas about Tanzanian TBSs, providing a preliminary investigation into the unsafe practices, strengths, and readiness to collaborate with allopathic medicine in order to increase access to quality orthopaedic care.

## Methods

**Figure 1:**
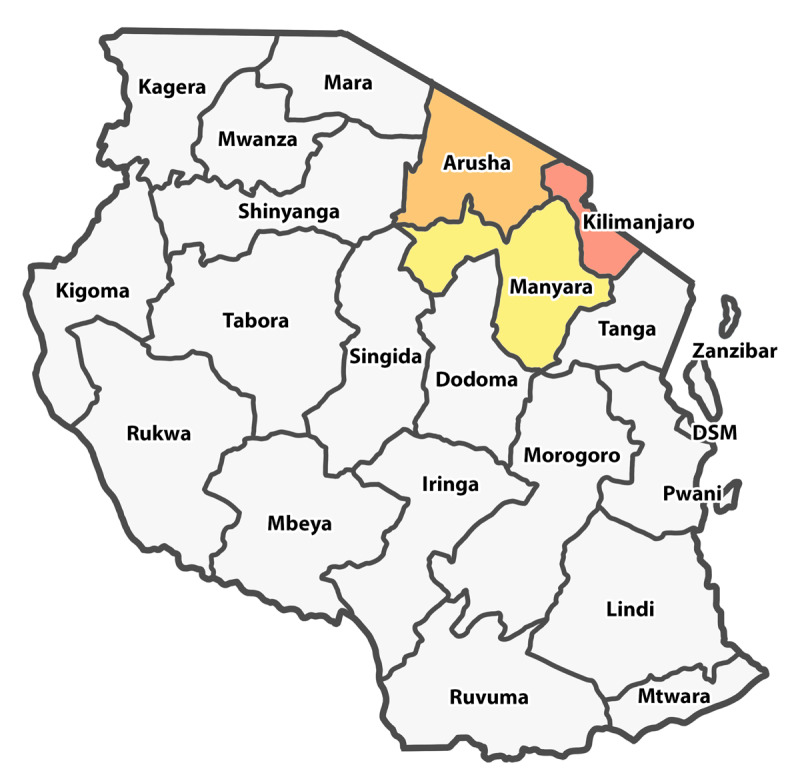
Map of Tanzania with Kilimanjaro, Arusha, and Manyara regions highlighted. We interviewed two traditional bone setters from each of these regions.

Between June and July 2017, we conducted semi-structured interviews with six self-described TBSs, two each in the northern Tanzanian regions of Kilimanjaro, Arusha, and Manyara (Figure 1). The TBSs sampled were considered to be key informants based on community perceptions and reputation.

TBSs were interviewed at either their place of work or their home. The interview teams consisted of two to four people, always including at least one foreign and one native investigator and up to two foreign and two native investigators. Interviews were conducted in English by a foreign investigator (EBC) and live translated into Swahili or M’aa by a native investigator (MM or HM). Both native (OS) and foreign (JEO) contextual note takers were present during interviews. Interviews lasted between one and two hours and were semi-structured around the topics of background, practices, and views on allopathic medicine for musculoskeletal injuries. Audio and video were recorded for later analysis.

We transcribed *verbatim* each interview and one investigator (MM or OS) subsequently translated them into English [26]. A foreign investigator (EBC) analyzed the interviews in NViVOv.11.0. using a deductive qualitative coding method [27]. Transcripts were coded using both pre-defined codes based off the interview questions and open-coding to incorporate practices that surfaced spontaneously during conversation. Codes were combined into categories and charted onto a matrix along with notes for comparison and generation of descriptive summaries.

Researchers verbally obtained consent to interview prior to the day of interview and written on the day of the interview. Written consent was obtained by initials or thumbprint depending on literacy. Institutional board review was obtained from Tumaini University and Kilimanjaro Christian Medical College (No. 2220).

## Results

### Demographics

The TBSs were all men aged 45 to 69 years. The majority identified as members of the Maasai tribe, while others were of the Sambaa and the Jaluo tribes. Religions represented were traditionalist, Christian, and Muslim. The highest level of formal education was primary school, but most had no schooling.

Traditional bone setting was the primary source of livelihood for only one interviewee, while the rest supported themselves in other ways including selling medicinal herbs, other forms of traditional healing, and pastoralism. The TBSs had difficulty estimating monthly income due to inconsistent patient flow, and the Maasai pastoralist custom of receiving payment as livestock barter. For those who were able to estimate a number, incomes ranged between 200,000 Tanzanian Shillings (TSH)/month and 800,000 TSH/month ($86.76 United States Dollars [USD]/month and $347.02 USD/month).

All TBSs had been practicing for at least 20 years, and they approximated 2 to 500 patients per year. They reported patients are recruited by word-of-mouth and usually come from the TBSs’ surrounding areas.

Knowledge acquisition occurred through paternal inter-generational passage and through apprenticeship with male neighbors. One TBS had been setting bones in livestock for 10 years prior to establishing himself as a bonesetter for humans.

### Medical Practices

Extremities were reported as the most common body parts treated, but a variety of bones, including spines and clavicles, were also reported. All TBSs treated closed fractures, open fractures, and malunion, and some treated dislocations, chronic pain, joint issues, ligament and tendon injuries, and congenital malformations.

**Figure 2:**
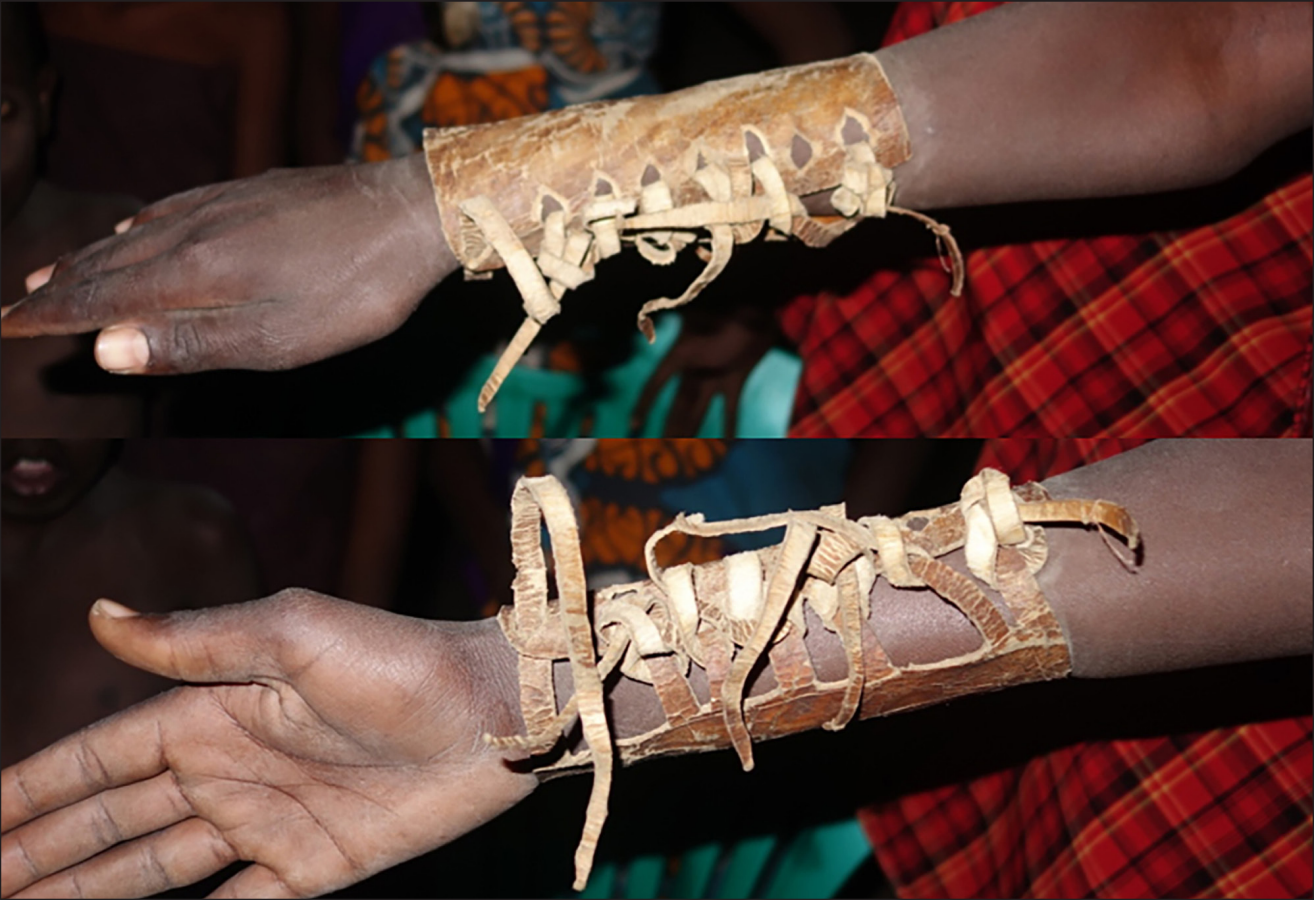
A model wearing an adjustable cow hide splint used by two of the Maasai pastoralist traditional bone setters.

Closed fractures were located via palpation and one TBS obtained radiographs. Reduction was achieved through massaging and manual traction. Bones were fixed by tying animal hide or pieces of wood around the affected body part and using clothing, pieces of discarded mattress, or pharmacy supplies as padding against the patient’s skin (Figure 2). Wood splinting was removed, cleaned, and re-applied every two days to two weeks or ‘whenever splinting becomes loose.’

Wound care for open fractures was handled with a combination of both allopathic and traditional techniques. TBSs used pharmacy-bought disposable gloves, topical antiseptic, and sterile cotton and gauze. Some boiled instruments in water for sterilization, sent patients to hospitals to receive tetanus vaccination, and collaborated with a medical nurse for sterile wound dressings and a medical doctor for antibiotic prescriptions. One reported learning sterile technique from government-organized trainings. Infection control techniques less familiar to an allopathic setting included topical and oral traditional medicines, wound desiccation, washing with saltwater, and suturing with unsterilized tree needles and cotton thread. TBSs also practiced puncturing or cutting intact skin with unsterilized tools.

‘I enter a finger into the wound to remove small bones which might have been left in the wound. After that I massage the protruding bone until it gets in its position, then I stitch the wound, but leave gaps to ensure that there is a place to put the herbs through…If the wound is small, then I will have to enlarge it myself with a knife so that I can get out the small pieces of bones…I don’t have [gloves] I just do it bare handed and afterwards I wash my hands.’

Pain control throughout fracture care was either left untreated or treated with either allopathic medicines like tramadol or oral and topical traditional medicines.

The TBSs reported treating malunion by breaking the healed fracture site using either their bare hands or a tool. There were different beliefs about how to prepare the site for the break. Some made the bone more breakable by first applying heat with fresh goat intestines or a hot machete while others believed the healed site is inherently more breakable and requires no preparation. During the breaking procedure, pain control was addressed with physical restraint or calling a medical doctor to administer local anesthesia. Only one TBS did not rebreak healed fractures, and treated malunion by leaving the affected body part in a cast made of tree sap.

No TBSs endorsed using spiritual treatment with their patients, but they did report personally praying to God for their patients’ healing or finding another person to pray for their patients. When asked specifically about animal sacrifices, one denied the practice calling it ‘witchcraft.’

Patients recovered either at their homes, the home of the TBS, or both. One TBS reported having a ward for patients in another region in the past. The TBSs each recommended a period of immobilization followed by exercise. They followed-up with their patients most frequently daily if patients stayed at their home to least frequently every two weeks. Patients were determined to be healed when they were pain-free with functional testing.

Services were bundled at a set price depending on injury location and type. No TBS increased their price if additional follow-up or treatment was needed. Patients often delayed payment due to inability to pay, but few absconded entirely without paying. Adults ranged in price from 170,000 TSH to 500,000 TSH ($73.74 to $216.89) and 50,000 TSH ($21.69) for a child. The three Maasai TBSs practicing a pastoralist lifestyle also accepted a cow or a calf for an adult and a sheep or a goat for a child. Other models of payment included paying based on satisfaction with care and ability to pay.

‘It depends on the patient’s financial status. The maximum I have received is 500 thousand shillings and for those who are poor, sometimes I don’t charge them.’

Most of the TBSs recognized some cases are beyond their capacity to treat and have referred patients either to another TBS or more often to an allopathic hospital. Cases that were referred include head, neck, and spine injuries, as well as severely comminuted fractures and gangrenous limbs. Only one TBS reported that there is no case he will not attempt to treat and has never referred a patient to another TBS or allopathic hospital.

### Perspectives on Allopathic Medicine for Musculoskeletal Injury

All TBSs interviewed believe allopathic medicine is competent in treating musculoskeletal injuries, but they did voice concerns about low quality of care and limitations in access. Common issues included infrequent follow-up and malunion as a result of hospital treatment.

‘The problem with the hospital is that once they put a patient in POP [plaster of Paris] they do not make follow-ups nor give any medicine. That is why when a patient who was once set in the hospital falls, there is a likely chance that the same place might break again.’

‘Some people in hospitals apply a full POP cast on the first day and schedule the patient for a follow-up clinic after six weeks when the swelling subsides and the POP has become loose and does not work well.’

‘There are several cases of people taken to [the hospital] and they had POP on their broken legs and they recovered, but the leg is no longer straight. The problem with the hospital is that they do not stretch and massage the patient’s leg. The patient might have overlapping bones due to the accident.’

‘I have seen many patients treated in the hospital and they have lame legs or hands, but for me, it has never happened that I treat a patient and the leg or hand bends.’

One TBS voiced concerns over the prohibitive expense for impoverished patients to receive care at allopathic hospitals. There were also concerns about mutual trust from both sides.

‘I do not completely trust the hospital and neither do my patients.’

‘[The hospitals] don’t trust me.’

All TBSs endorsed a willingness to collaborate and to undergo trainings in allopathic techniques for musculoskeletal care. Many said that the best way to improve musculoskeletal care in Tanzania would be through better communication between allopathic and traditional providers. They expressed interest in connecting with allopathic specialists to improve their own practices and wanted supplies such as anesthesia, X-ray, and allopathic splinting materials. Many also wanted help building their own wards in which their patients could recover.

‘There is a Maasai saying, “One pillar cannot support the house”…We [should] build unity between us and the hospital. If there is a case that the hospital cannot do they can consult me and if there is any case that I cannot treat then I consult the hospital.’

‘The best way is coming together, working together and identifying shortages in hospital procedures and in the traditional procedures.’

‘I want to be provided with equipment to replace the local ones I have like the hide…I want you to connect me with specialists in the hospital so that we work together and my work will improve.’

‘If I get someone to anesthetize the patient it will be a very good step because now what we do is the patient is held with strong men to prevent him from being uncooperative during the procedure because of pain.’

The TBSs also provided their own models for collaboration. One recommended that he would refer patients to a hospital for reduction and fixation after which his patients are returned to him for administration of traditional medicine and follow-up. Another recommended that TBSs are paired with hospital practitioners who can administer anesthesia for TBSs to manipulate fractures.

## Discussion

This study provides a foundational descriptive understanding of traditional bone setting practices and perspectives in northern Tanzania with which to assess safety concerns, strengths, and readiness to collaborate with allopathic providers. The TBSs we interviewed practiced unsafe techniques such as splinting without using radiographic imaging putting patients at risk for malunion or nonunion and manipulating or creating wounds with unsterile tools putting patients at risk for further unnecessary injury and infection. Their strengths include the advantages of effective word-of-mouth communication networks, geographic accessibility, and financial affordability. Past experience referring complicated patients to hospitals and collaborating with allopathic providers, as well as interest in collaboration demonstrate the readiness of these TBSs to work more closely with allopathic healthcare through training and referral systems.

Previous studies on traditional bone setting have primarily come from Nigeria. In comparing the TBSs interviewed for our study to that of Nigeria, we discovered Tanzanian TBSs had similar backgrounds including limited formal education and the passage of knowledge through male generations [4,12,13,28,29]. Similarities in practices included palpation for location of fracture, reduction through massage and pulling, fixation with wooden splints, the use of herbal traditional medicine, and the use of allopathic techniques such as X-ray [4,12,13,28,29]. Studies out of Nigeria have criticized the spiritual and superstitious components of traditional bone setting, such as animal sacrifices and incantations, but we did not find any Tanzanian TBSs who used spiritual or superstitious healing [4,12,28]. It is impossible to extrapolate a complete lack of spiritual practices among TBSs in Tanzania, especially since spiritual practices have been endorsed by modern Tanzanian traditional medicine practitioners [20,30,31]. However, it is important to note the difference in political contexts for traditional medicine between Nigeria and Tanzania. Nigeria welcomed traditional medicine legally in the 1960s and currently has laws protecting citizens against accusations of witchcraft whereas Tanzania has a long history of anti-witchcraft laws that continue to the present day, contributing to stigmatization of traditional medicine [1,30,31,32]. In our interviews, one TBS even referred to animal sacrifice with the deeply politicized and stigmatized word of ‘witchcraft’ [30].

Prior publications on traditional bone setting in Sub-Saharan Africa report the complications of traditional bone setting practices as a threat to public health and have fueled efforts to train TBSs [2,4,9,12,13,14,15,28]. These complications include malunion and nonunion from lack of radiographic imaging or proper reduction, compartment syndrome and gangrene from constrictive immobilization, and infection and tetanus from lack of sterility, lack of prophylaxis, and scarification [14,29,33,34,35]. Our study identified similar safety concerns amongst the practices of Tanzanian TBSs that need follow-up quantitative research to determine the degree of threat to the public.

The majority of the TBSs interviewed in this study did not use radiographs or other imaging modalities to locate and characterize fractures and assess healing, concerning for nonunion and malunion. However, unlike studies reporting a complete lack of attempt at reduction, all the TBSs in our study reported attempted reduction [33]. The use of adjustable animal hide splinting and frequent untying and adjusting of wooden splints could theoretically prevent complications due to vascular compromise; however, adjusting the splints may increase the likelihood of failed reduction and resultant malunion or nonunion. Infection is also a concern with the TBSs’ practices. While the majority used some aspects of sterile technique for infection control, we identified techniques with high risk for infection, including reducing open fractures with bare hands, scarification, and cutting into skin with unsterilized tools. Another unsafe practice is the use of blunt trauma to rebreak malunited fractures, which could cause unintended injury to the healed site and surrounding tissue. Furthermore, without radiographic imaging, assessment of malunion relies on clinical judgement, which could be prone to error by subjectivity.

Patients face many barriers accessing allopathic orthopaedic care in Tanzania. One such factor is the low availability of orthopaedic care. According to one source, there are 45 orthopaedic surgeons in the country, totaling to about 1.4 orthopaedic surgeon per one million Tanzanians [23]. Traveling to urban centers where orthopaedic surgeons operate can also be an obstacle. In 2013, 19% of regional roads and 2% of district roads were paved in Tanzania [36]. Considering that in 2011 to 2012, 28.2% of the population of Tanzania lived below the national basic needs poverty line (TSH 36,482 or $15.81 per adult per month), poverty may also make hospital transportation and treatment costs prohibitive for many patients [37].

While the capacity of orthopaedic care continues to grow, traditional bone setting offers a more immediate solution for patients seeking treatment for musculoskeletal injuries. Our interviewees practicing in rural communities were more easily accessible to patients than the nearest hospitals. One described his remote practice as the sole source of care for his neighborhood. To reach him from the nearest city with a hospital that provided orthopaedic care, our team travelled nearly 100 km via a long-distance bus on paved roads, a local bus on unpaved roads, and a motorcycle taxi on unpaved roads to reach his place of practice. Nearly hundreds of patients per month were acquired through word-of-mouth communication both within and outside of their immediate communities, demonstrating the trust the patient population has in TBSs and in the effective communication networks already in place for patient flow to these rural practitioners.

Due to greater accessibility, traditional bone setting has the ability for more frequent follow-up visits. The TBSs in this study visited their patients as often as every day when patients stayed with the traditional practitioners and still frequently even after patients returned to their homes. Frequent follow-ups can ensure strength rehabilitation and catch warning signs of complications like infection for quick referral.

Cost is another advantage to traditional bone setting. The average cost of a hospital admission for orthopaedic surgery at KCMC is about 500,000 TSH or $216.61, which is the maximum we found in our interviews (KCMC Orthopaedic Ward Billing Book 2017). The cost of traditional care is bundled in one lump sum and additional costs due to poor outcomes are incorporated up front. At KCMC, each additional day adds to the cost the patient must pay sometimes compounding into enormous sums that the patient cannot afford resulting in extended hospital stays. Furthermore, TBSs may be more affordable for specific minority tribes. The Maasai of Tanzania and Kenya are well-known for their adherence to the traditional lifestyles practiced by their ancestors for generations, including the use of livestock in trade. Accepting livestock in exchange for treatment may be more affordable for monetarily impoverished yet resource rich Maasai patients, making care available to patients that would otherwise be unable to pay for treatment with cash.

Agarwal et al. states that in order to successfully incorporate bone setting into health systems, there must be strong commitment by the government, allopathic providers, and the TBSs themselves [2]. Our study found at least some degree of commitment expressed through interest to learn allopathic techniques and establish lines of communication with hospitals as well as previous experience working with doctors, nurses, and pharmacists. One TBS had undergone government-organized trainings where he learned the sterile techniques he utilized in his practice, indicating it is possible to successfully train TBSs to practice safer techniques.

All TBSs in this study expressed willingness to receive training and access allopathic techniques such as X-ray, anesthesia, and improved fixation materials. Two even gave potential models for collaboration indicating a deeper contemplation of working together with allopathic providers. The model describing a mutual referral system for treatment and recovery discards unsafe practices while incorporating the advantages of bone setting, such as effective communication networks penetrating largely inaccessible geographic areas and the ability for frequent follow-up. All but one TBS recognized limitations in their abilities and have referred patients to allopathic hospitals. Such behavior demonstrates that a referral system could be designed so that TBSs are treating simple, closed fractures thereby reducing burden on hospitals and referring complicated fractures that are beyond the capacity of traditional care.

In order to harness the interest we found for collaboration and ensure cooperation, respect from the allopathic community for the work of TBSs is of utmost importance. The comment, allopathic medicine ‘does not trust me’ has been reflected in studies surveying allopathic providers about their opinions on traditional medicine [38,39]. Training should meet TBSs at their knowledge levels and provide information deemed useful by TBSs so as to not risk demeaning and alienating them [38]. The planning of training expansion should involve TBSs, taking advantage of their perspectives to avoid failure from paternalism, and their strengths like communication networks to penetrate into rural areas and unionize remotely located TBSs. In fact, Tanga AIDS Working Group, a successful Tanzanian collaboration between traditional practitioners and allopathic providers, attributes its success in part to being a traditional practitioner-lead initiative [40].

In order for traditional care to gain respect and promote relationship building with the allopathic community, further research should be done to quantify the contribution of bone setting to alleviate the musculoskeletal disease burden in Tanzania. Quantifying successful treatments and complications would also help inform trainings to target specific practices and populations of TBSs. Such a study would need the cooperation of TBSs and may be difficult to implement due to the lack of documentation by TBSs and unwillingness to admit to complications. During our interviews, TBSs gave anecdotes about successful treatments, but did not report any negative outcomes. Traditional medicine in Tanzania is a competitive market and traditional practitioners advertise with testimonials, which could lead to resistance to expose failed treatment [20].

This potential for biased reporting is the greatest limitation for our study. The majority of the interviews took place in public areas with many community and family members present; ideally, the interviews would have taken place with more privacy to encourage honesty. Furthermore, the presence of the foreign and native allopathic healthcare providers and investigators during the interviews could have instigated social desirability bias leading to falsified enthusiasm to collaborate.

Future studies should also include an inductive framework analysis of this data performed by both a cultural insider and outsider to determine conceptions of health and healing that would guide TBS training and outreach design. Lastly, an investigation into perspectives of the allopathic orthopaedic community on collaborating with TBSs is necessary to determine the feasibility of teamwork between traditional and allopathic providers.

## Additional File

The additional file for this article can be found as follows:

10.5334/aogh.2878.s1 Tanzanian Bone Setter Interview Transcripts. Raw transcripts for interviews with traditional bone setters from June to July 2017. Includes M’aa and Swahili as well as English translations.
