# Supplementary material for: Practices and Perspectives of Traditional Bone Setters in Northern Tanzania
Source: Ann Glob Health. 2020 Jun 16;86(1):61. doi: 10.5334/aogh.2878 (PMC7304448; doi:10.5334/aogh.2878)
Supplement: Tanzanian Bone Setter Interview Transcripts. — Raw transcripts for interviews with traditional bone setters from June to July 2017. Includes M’aa and Swahili as well as English translations. [file agh-86-1-2878-s1.pdf]

TBS 1: Moshi

Interview conducted in English and Maa, translated and transcribed my ML

MLM: Ejoito toi aikioto duo pee kingilikwanishore iyie amu kidol duo aajo ore eng'en niata oo enaimaki iltung'an, naa eng'eno nagut oleng naretokito iltung'anak oogila neeku ninye pee kietuo akilikwanishore iyie

RESP Meata shida amu aidipa oshiake iltung'anak oora oong'wan aikilikwanishore. There is no problem because have already been interview for four people.

MLM: Kamaa eton itu kipuo dukuya, iata oswali lingilikwan iyiok? Do you have any question before we proceed?

RESP: Maata . I don't have

MLM: Kanyoo engibiribirata ino edukuya enidol ilaikeernok kiponu aikidikwanishore? What are your first thoughts if you are firstly approached by researchers?

RESP: Meata amu ore pee elotu ilo lee pee aikilikwan tene nanyor naleng amu adolita iltung'anak kumok eimuia nepuo sipitali hata tenebak sipitali kulo tung'wanak esidai neton ake mayamini esidai ajo aidim ataretoki. It is really good, whenthat man firstly approached me i become glad because this will make the hospital to trust me because they do not trust me.

MLM: Toliki duo iyiok ajo kakwa ramatat oshi injo iltung'anak? Tell us those services you provide to your patients?

RESP: Kumok amu ore ninye kuna moyiaritin seroi aitau nena tukul, enaa enamuratuni naa ninye enoshi namutita iltung'anak amu akemit asarge tosesen nepurd elewaishu, naidikidik ake iloik. There many because there are other diseases like "enamuratuni" enamuratuni one of the diseases that make people emmaciated and caused inflamation in the genital organs. Also i am a bone setter.

MLM: Kamaa duo kaa ramatata injo iltung'anak oogila? How do you treat people with broken bones?

RESP: Ore oltung'ani oiwuang'a engunja nashor aitorioo, ore enaa iloik oiperare naidikidik naa enjoni enger nashal aidikidikie naa ingiri naatoito enya ilo tung'ani. For a person with a broken leg I massage it and correct the mislocation of bones then i tied using a fresh sheep hide. The patient food is dry meat.

MLM: Kamaa taata oltung'ani otigile temusano neeku etoriwie engeju arashu engaina iramat? Do you also treat long time injuries that had made the arm or leg lamed?

RESP: Eee ore oltung'ani lijo ilo naa akagil nanu naitoki aidikidik, kake ore oltung'ani otigile engoriong naa akaitorioo ake neendelea oltung'ani aaok ilo shani oo nena kiri naatoito. For such a case i will have to break the lamed part and join it afresh and for the back bone I just massage it and the patients will continue to take the herbs until he/she recovered

MLM: Iltorioo ingera naariwuo ingejek? Do you treat bones in infant babies?

RESP: Eee tenemutu eton akinyi naitorioo kake tenebulu nemaidim. Yes i can do it at a tender age but when they become old then i can't do it.

MLM: Kamaa kenasiai ake indegemea enake iata engai siai? Do you only depend on this work or you have other sources of income?

RESP: Enanoto lelo tung'anak naa aang oshi nanu aya nalo aramat neeku ingaramani ake kiponu awoshokino. Kake ele kasi loolkeek aitegemea oleng. I depend mostly in selling herbs and if the patient are brought to me i treat them and charge.

MLM: Kamaa kulo keek iloomoyiaritin pookin enake iloonguti moyiaritin pookin? Do you have herbs for all the diseases?

RESP: Etii ilomoyiritin pookin. Yes there are herbs for several diseases.

MLM: Kengidikidikore oo iloik ninye integemea naleng? Do you depend much on bone setting?

RESP: Meeninye amu metii ina anake ore saa nitu atum oltung'ani lataramata nipidi pee asotie ingera ena siai oo ilkeek. No because the patients are not many.

MLM: Kamaa taata tiatua ewiki keya nitum iltung'anak aja itum tiatua ewiki? How many patient do you receive in a week?

RESP: Ore apa tenda wiki nashomo mererani aidikidik oltung'ani otigile. Kake maidim atejo keltung'anak aja atum tewiki amu eton mayeloi naleng. I can't tell how many in week. Last week i got one in mererani.

MLM: Kai toi ingo enitum iltamweiyia? How do you get patients?

RESP: Oltung'ani laayiolo ninye layaki iltamweia arashu elatia arashu oltung'ani laidipa aataramata. I get patients from people who knows me, from neighbours or the already treated patients by me.

MLM: Kamaa ena siai kanu interua? When did you started bone setting?

RESP: Aata ake ilarin ishirini nane amu aikigira aas oo papa neton ake eesita papa kake etamorua oleng. Have like 28 years because I started working with my father. My father is still doing it but he is old now.

MLM: Kamaa teleari keltung'anak aja itaramata? How many people did you treated this year?

RESP: Iltung'anak osom oo imiet tolari. About 35 people in a year.

MLM; Kamaa indim aatoliki iyiok eningo enibak oltung'ani otigile? Can you tell us how you always treat a broken person?

RESP: Ore tanakata enayauni oltung'ani otigile naa aang ake aya nalo aing'oru enger, neyeng'i nitauni eilata pee esihoru eilata aidipa ateena. Nadumu inashoni enger nairin tukul nalo oserok ayau ilkeek neyiarakin neeku ninye ook eneengare, neaku kuna kiri naatoito ninye aitumia. Neeku enadapana ake enger eyeuni, oo ena ilata, oo kuna kiri naatoito. When the patient is brought to me, I just find a fat sheep slaughter it and the oil is made. The hides is used to tie the broken part after massaging. I go to the bush to look for herbs and the oil is given to the patient and the dry meat.

MLM: Kejaa iloshani liyiaraki ele tamweiai? What is the names of the herbs?

RESP: Eji iloshani oshungwai naa oldoinyo etii. It is called oshungwai and it is found in the mountain.

MLM: Kamaa enekiakini oltung'ani otadanya oloito enjoni kaja ingo eniramat? How do you treat a patient with a protruding bone outside the skin?

RESP: Akarinyoki ake oloito atua natumoki airina, akashor ake oo matadua ajo etushukokine oloito atua. I just massage it until I see that the protruding bone is in its position.

MLM: Kanyoo toi ishorie oltungani? What do you use to massage the patients?

RESP: Ingai ake nemeeta endoki naishop toongaik. I use bare hands.

MLM: Kengata nabaa eya enishor oltung'ani? How long do you massage the patient?

RESP: Eya engata amu eya ninepu etapaashate iloik neya netararate iloik. Kake tenitu egila ainyalakino neya engiti kata eya. It can only take long if there is a seriuos dislocation or if there is a serious grinding of bones.

MLM: Kengata nabaa eya eninyalakino oltungani engilata, kengata nabaa ishor? How long does it take to massage when the dislocation is abit serious?

RESP: Tenetigile ainyalaki neya saa oo enusu, kake tenitu egila ainyalaki neya esaa nabo ometaba niindik kake mbaka taa niindip aateena. When the dislocation is seriuos then it takes more than one and half hour, but normal takes less than that.

MLM: Oo tenetapaashate iloik kake itu edanya enjoni kai ingo? What it if there is a dislocation but no brotruding of the bones outside the skin?

RESP: Airapirap ake nishor omenototo iloik kake mekidanya enjoni. I just massage until the bones come together but we can not cut the skin.

MLM: Kamaa ina ker nayieng'i kang'ai naishoru? Who bring the sheep to be slaughtered?

RESP: Lelo tung'anak ake oinyan'u arashu eya nainyang'u neponu aalak. The relatives of the patient, or sometime I might buy it and they pay back to me.

MLM: Kanyoo pee eyeng'i enger? Why do you slaughter a sheep?

RESP: Eyeng'i naatoi enger amu kiyeu enjoni, nekiyeu ena ilata naok andamweiai, nekiyeu kuna kiri naatoito amu ninye endaa nanya ele tung'ani. Because we need the hide, the oil and the dry meat as food for the patient.

MLM: Kamaa enashoni nashal kaa hoto napetaa osesen? Which side of the hide come into contact with the body?

RESP: Enahoto nashal ninye itepetaari amu enashoni nashal nakurrakino osesen nepik engima nisho lelo oik midikidikoto amu enipik sementi nepik engirobi iloik. The inside part of the hide, because it provide the broken part with warm temperature.

MLM: Kamaa taata eneeku etadanye enjoni nejing engidonda meitong'wa enashoni inakidonda? Does the wet hide totten the wound in the broken part?

RESP: Ore enekingo aiking'asa aalepu osarge arashu olpile nekintoi pii nekitumoki ateen tina shoni nashal, nemitong'wa pii ina kidonda. What we do is that we clean the wound and make sure that it is dry before we tie with the wet hide and it cannot rot.

MLM: Kaja ingo eniisuj enakidonda keeta endoki nisuje enakidonda meitau ele sarge arashu ele pile? What do you sue to wash the wound?

RESP: Eeti tukul etii olnjani oji oltemuai naa ninye oshi kisuje ina kidonda amu iitoi nemitiki engidonda eng'wei. There is a herb known as "oltemwai" and it is the one we use to clean the wound and it prevent microrganism from rotting the wound.

MLM: Kai oshi itumiemie oltemwai? Where do you get oltemwai?

RESP: Etii angalo inewei nekimanya. It is just near our home

MLM: Kamaa toi eniyen ene netigile ipik ingeek? Do you cover the wounds with woods?

RESP: Mepiki ayiolo taake ina kake mapik oshi nanu amu meibung' iloik esidai naa eimisa oltung'ani engeju. No i know that it can be used but I realized that the patient bones will not recover well if you use the woods so i don't use.

MLM: Kanyoo tooshi ianikinyenye enashoni engeju? What do you use to tie the hide on the broken arm or leg?

RESP: Ore toi ena shoni aiboru enaa engeene kake ore enashoto naiteru naa ninye endapash nindorongen engai shoto nirinie engeju esidai. You know the hide is made in way that one side is larger and it bicome thinner as you apprach the other end. This will enable you to tie the arm with the same hide.

MLM: Kamaa eniindip ataramata oltung'ani kengata nabaa eya itu igut? How long does the patient remain imobile after treatment?

RESP: Ore naatoi teyeok ore inaaji natii ele tung'ani naa akinoki engima naleng pee etum oltung'ani engima. Kake eng'as ake oltung'ani abik ewiki nabo itu igut, kake eeta sii intriman enyana naalotie. Ore tenelusoo ewiki neas mazoezi. Naa nanu kenya ake olotu aalaku. In the house where the patient is, we light fire so as to warm the house. The patient remains imobile for a week and he/she has his/her walking sticks to walk with it after a week where he does exercise.

MLM: Kengata nabaa eya oltung'ani omishiu pii nilotu alak? How long does the patient take to recover?

RESP: Iwikii ong'wan ake matejo alapa obo ake. Only four weeks, let us say a month.

MLM: Kamaa enishiu oltung'ani nelo aang, injo ilkeek oya alo aok? Do you give the patients herbs to use at home after you rpoved that he has recovered?

RESP: Aaa meeta ilkeek laitoki aisho akelo ake, meteleku eneeta oltung'ani engai nyamali. No more medication expect when the patient has other problems.

MLM: Kamaa taata kai ingo eniramat oltung'ani oishiwuo kake itu apa idiki esidai? What do you do to a patient who has recovered from a wrong bone setting?

RESP: Akagil negilai kirahisi amu ore inewei netigile naa akejing alaishimi metaa eniosh ninye aikonji negila, ore pee egila niingil taata iyie aidikidik, ore ninye ilo laa akipere apa oloito, eniosh ake sii ninye nedar inewei niingil ake aidikidik. I just broke the place and reset again. You know the proken place if wrongly join will join weakly and can be broke easly too.

MLM: Kanyoo toi igilie? What do you use to broke the bone?

RESP: Egil toi engaina, enitashaki ake duo taata ewei niosh tengaina negila. Even the puch by hand can broke the bone.

MLM: Iata onjani linjo pee mening emion? Do you have anasthetic?

RESP: Maata nanu olnjani akeng'iri ake oltung'ani? I don't have

MLM: Keya nidung arashu ing'am enjoni pee ejing onjani? Do you sometime make a hole or a mark over the broken part for easy medication?

RESP: Madung, nemang'am kake tenibulubul tewei naa akaud ake tosindano nalepu ilo pile napik oltemua atumia ilo tidu amu kelepu oltemwai ilo pile pii. I do not cut but when the area develop some blisters then i can remove the blisters by using and needle then I put oltemwai using the same needle.

Iasita toi enasiai enaa oloiboni, enake alaomononi? Do you perfome this treatment as a religious healer?

Mara kake ore tenelotu altamweiai naomon Enkai pee eshiunye lelo keek lasho. I am not but i always pray God to make my herbs curable to my patients.

Kanyoo oshi gharama ena ramatata ino, imbisai aja indai oltung'ani eniindik? How much do you charge for this treatment?

Elaki oo sabini kake elak ingaramani naitumiayie, enainyang'ua enger nelak. 170,000Tshs but he reambance the cost. For example when I buy a sheep , the patient has to pay.

Kakwa oitin osesen naleng itaramata oleng? Which part of the body have you treated mostly?

Ingejek ake,oo ingaik, oo iltung'anak oiwuang'a engunja amu eeta ninye engunja ilkeek oong'amakini. Only legs and hands and people with dislocation in the hip bone.

Iramat oshi iltung'anak oiwuang'a imorlooshi arashu impit oo ingejek? Do you correct people with broken cartilages?

Ashor oshi sininye nang'amaki alnjani. I just massage, cut the area alitle bit and put a medicine

Kamaaa oltemwai kaindong paake ipik enake? Do you grind oltemwai before puting to the wound.

Aketiu toi oltemwai enaa eilata neeku aipik ake ene neya oltung'ani ayelie. The oltemwai looks like oil so you just rub it on the wound.

Kamaa taata kerisio gharama eniindik engaina arashu engeju arashu emorloo? Is the cost of setting legs, arms and cartilages the same?

Erisio kae ghrama elaki ake oo sabini. It is the same 170,000Tshs

Kai taapa inguna oo tayelo enasiai? How did you first learn this treatment?

Papa ataduaka. I immetated from my father.

Ketii likai tengang inyi oyiolo enake iyiek otusuja papalino? Is there any other relative or brother or sister of yours who also immitated?

Metii likai nanu ake. No one elso it is only me.

Irara aja? How many are you in the family?

Kira imiet we are five children.

Paa ketejo sii ninje lelo ijaribu nelaikino enake itu eng'asa aatem? Did the rest tried and fail or they had never tried?

Itu eng'as aatem. They have never tried

Kamaa taata kengata nabaa toi kinteng'ena papa lino? How long have learned from your father?

Aiteng'ena ilarin okuni. I learned from him for three years.

Kaikinteng'ena toi enake aitaduaka ake? Did he teach you or you immitated?

Aiteng'ena amu eneyakini iltamweia naa nanu ejoki iyakaki olnjani oje, imbung'aki ene, iyakaki endoki naje oo matayiolo sinanu. He teach me as well as I immitated. I was with him during treatment and he ask me do this do that and finally I learn a number of things.

Keas ninye yeyelino ena siai? Does your mother do this treatment?

Meas ninye yeyo enasiai. No

Keas apa kuyiaa lino ena siai? Was your grand pa a bone setting specialist?

Eee eas apa naa ninye apa oiteng'ena papa. Yes he was doing it and that is where my father immitated.

Inoto aikata ewei nekitorikoki aiteng'en saidi imbaa ena siai? Have ever received any training about your work?

Itu aikata nanu atum engiteng'ena enasiai tiai wei. I have never received any training.

Kamaa taata enijur huduma esipitali oo ena oo ilmaasai kanyoo indim atejo. What do you recoment if you look at the hospital treatment in bones and the treatment you provide as traditional bone setters?

Sidan pookin neesita pookin endoki sidai kake kijo tofauti amu enekiindip sipitali atipik ena sement enye neemeta engai uduma nekinjo nemekisujaki ninje, naa ina pee ore oltung'ani odikidikoki tesipitali enebatata penyo nelelek pee itoki agila. Kake enekindikidek iyiok nekinjo ilkeek nekisujaki? They are all very good and they are doing something good. The problem with the hospital, once they had put a patient a POP they do not make followups nor giving any medicine. That is why when a patient who was once set in the hospital fall there is a likely chance that the same place might broke again. For us we make followups.

Kamaa oshi eneim iwiki ong'wan nishiu oltung'ani iltoki ake asujaki? Do you still make followups after the patient has fully recovered?

Maigil asujaki amu eeku ishiwuo. No I will not make followups

Keeta toi engoitoi esipitali niyiolo enake arashu nintumia? Do you use or know any of the hospital procedures in your treatment

Maata tukul. No at all.

Ketii ingulie tokitin nemindiim ataramata? Is there any other treatments in bones that you can't do?

Etii amu ore iloogila ingoriong'i oo ilukuny. Yes. I cant help people with broken backbones and heads.

Keton ake iasita esiai oo papalino? Are you still working with your father?

Etii ake ioitin neas openy naasisho sinanu openy kake eya naipotu enetii ewei nemiidim aidiko amu etamorua. There some treatment every body perfom individually but he may call me if there is something he can do because of the old age.

Kamaa taata ketii aikata oltung'ani lekiakaki nijoki ewaita sipitali amu maidimu? Have you ever refered a patient to the hospital?

Aaa amu ore ilainoto naa kulo loongejek ake oo ingaik. No because I have been receiving arms and legs cases.

Kamaa naa nena weitin nijo miindim ataramata kai ingo? What do you do to those patient you cant treat?

Ore ilo lemaidim ataramata najoki ewaita sipitali amu etii alaipotokoki temererani naa emurt natarare najoki ewaita sipitali amu maidimu. For those that I can not treat I order his/her relatives to take him/her to the hospital. There was one patient with a broken neck and they brought him to me, I fail to treat and ask them to take the person to hospital.

Kamaa enekiunganishakini ildakitarini lesipitali pee itum airriwaki iltung'anak lilaikino kidimai ina? Is it possible to for you to be connected to the hospital doctors so that you can refer complicated patients?

Idimai tukul metii enyamali. Yes there is no problem it is possible

Iruk pee kindimieki pee iyiolou ataramatishore ingulie oitoi esipitali? Do agree to be tought hospital techniques that you will be using in your work?

Meeta enaany amu eme Enkai ninye naitutumo iyiok, naa naboishu esidai amu eji toi apake “meidimu eng'ape nabo engaji”. There is no problem about that because the knowledge I have is God's gift, and unity is good because the masai wise word say “ One pillar can not support the house”

Kamaa taata kanyoo ilimu nai naidimi ataas pee iboreshai eramatare esipitali oo ena ino? What should be done to improve hospital treatment on bones and your own treatment on bones?

Pooki nanu enajo injoo enesheti naboishu neeku eneyai oltung'ani sipitali ore pee epuku naishori mashomo aaramat, arashu enedol sipitali endoki nemidimu naaliki ore pee adol endoki nemaidimu naliki sipitali. The only thing to say is that it is better we build unity between us and the hospital. If there is a case that the hospital cant do they can consult me and if there is any case that I cant treat then I consult the hospital

Keeta engai toki niyeu niliki iyiok? Is there any thing else to tell us?

Meekure etii. No

Kamaa keya netii emweyian nashepu oltung'ani otigile? Is there any other side diseases for a pattient with broken bones?

Metii . No

Keya niata olnjani oretoki oltung'pee mitoki aning emion ebakitai? Do you have ansthetic?

Maata kake ore oltung'ani oning'ito irubat arashu osesen eme (naa ninye oshi kijo oltambasi) nekinjo olgilai. I dont have but for a person who has joint pain and legs pain (we call it tambasi) we give him/her olgilai.

Kaakeoki olgilai? Is olgilai taken by drinking?

Akeoki, akeyieri neoki. Yes it is boiled and drinked

Kai toi etumieki olgilai? Where do you get olgilai?

Intimi ake etumieki kake elakwa naleng. It is found in the bush far away.

Keata endoki nitu kingilikwanu? Is there anything we did not ask which you think that it important to ask?

Meeta tukul. no

Keeta endoki nijoki iyiok kias pee kimboresha? What do you recoment so that to improve our research?

Meeta kake ajo napai teneeku indipa nekishukoki inatoki ninoto. I dont have anything to recoment but I request for feedback at the end of the research.

Demographic information removed

TBS 1: Moshi, ENGLISH ONLY TRANSCRIPTION

Interview conducted in English and Maa, translated and transcribed by ML, additional transcription of English by EBC

RESP There is no problem because have already been interview for four people.

MLM: Do you have any question before we proceed?

RESP: I don't have

MLM: What are your first thoughts if you are firstly approached by researchers?

RESP: It is really good, when that man firstly approached me i become glad because this will make the hospital to trust me because they do not trust me.

MLM: Tell us those services you provide to your patients?

RESP: There many because there are other diseases like “enamuratuni” enamuratuni one of the diseases that make people emaciated and caused inflammation in the genital organs. Also i am a bone setter.

MLM: How do you treat people with broken bones?

RESP: For a person with a broken leg I massage it and correct the dislocation of bones then i tie using a fresh sheep hide. The patient food is dry meat.

MLM: Do you also treat long time injuries that had made the arm or leg lamed?

RESP: For such a case i will have to break the lamed part and join it afresh and for the back bone I just massage it and the patients will continue to take the herbs until he/she recovered

MLM: Do you treat bones in infant babies?

RESP: Yes i can do it at a tender age but when they become old then i can’t do it.

MLM: Do you only depend on this work or you have other sources of income?

RESP: I depend mostly in selling herbs and if the patient are brought to me i treat them and charge.

MLM: Do you have herbs for all the diseases?

RESP: Yes there are herbs for several diseases.

MLM: Do you depend much on bone setting?

RESP: No because the patients are not many.

MLM: How many patient do you receive in a week?

RESP: I can’t tell how many in week. Last week i got one in mererani.

MLM: How do you get patients?

RESP: I get patients from people who knows me, from neighbours or the already treated patients by me.

MLM: When did you started bone setting?

RESP: Have like 28 years because I started working with my father. My father is still doing it but he is old now.

MLM: How many people did you treated this year?

RESP: About 35 people in a year.

MLM; Can you tell us how you always treat a broken person?

RESP: When the patient is brought to me, I just find a fat sheep slaughter it and the oil is made. The hides is used to tie the broken part after massaging. I go to the bush to look for herbs and the oil is given to the patient and the dry meat.

MLM: What is the names of the herbs?

RESP: It is called oshungwai and it is found in the mountain.

MLM: How do you treat a patient with a protruding bone outside the skin?

RESP: I just massage it until I see that the protruding bone is in its position.

MLM: What do you use to massage the patients?

RESP: I use bare hands.

MLM: How long do you massage the patient?

RESP: It can only take long if there is a serious dislocation or if there is a serious grinding of bones.

MLM: How long does it take to massage when the dislocation is a bit serious?

RESP:When the dislocation is serious then it takes more than one and half hour, but normal takes less than that.

MLM: What it if there is a dislocation but no protruding of the bones outside the skin?

RESP: I just massage until the bones come together but we can not cut the skin.

MLM: Who bring the sheep to be slaughtered?

RESP: The relatives of the patient, or sometime I might buy it and they pay back to me.

MLM: Why do you slaughter a sheep?

RESP: Because we need the hide, the oil and the dry meat as food for the patient.

MLM: Which side of the hide come into contact with the body?

RESP: The inside part of the hide, because it provide the broken part with warm temperature.

MLM: Does the wet hide totten the wound in the broken part?

RESP: What we do is that we clean the wound and make sure that it is dry before we tie with the wet hide and it cannot rot.

MLM: What do you use to wash the wound?

RESP: There is a herb known as “oltemwai” and it is the one we use to clean the wound and it prevent microorganism from rotting the wound.

MLM: Where do you get oltemwai?

RESP: It is just near our home

MLM: Do you cover the wounds with woods?

RESP: No i know that it can be used but I realized that the patient bones will not recover well if you use the woods so i don’t use.

MLM: What do you use to tie the hide on the broken arm or leg?

RESP: You know the hide is made in way that one side is larger and it becomes thinner as you approach the other end. This will enable you to tie the arm with the same hide.

MLM: How long does the patient remain imobile after treatment?

RESP: In the house where the patient is, we light fire so as to warm the house. The patient remains immobile for a week and he/she has his/her walking sticks to walk with it after a week where he does exercise.

MLM: How long does the patient take to recover?

RESP: Only four weeks, let us say a month.

MLM: Do you give the patients herbs to use at home after you proved that he has recovered?

RESP: No more medication except when the patient has other problems.

MLM: What do you do to a patient who has recovered from a wrong bone setting?

RESP: I just broke the place and reset again. You know the broken place if wrongly join will join weakly and can be broke easily too.

MLM: What do you use to break the bone?

RESP: Even the punch by hand can break the bone.

MLM: Do you have anesthetic?

RESP: I don't have

MLM: Do you sometime make a hole or a mark over the broken part for easy medication?

RESP: I do not cut but when the area develops some blisters then i can remove the blisters by using and needle then I put oltemwai using the same needle.

MLM: Do you perform this treatment as a religious healer?

RESP: I am not but i always pray God to make my herbs curable to my patients.

MLM: How much do you charge for this treatment?

RESP: 170,000Tss but he reimburse the cost. For example when I buy a sheep , the patient has to pay.

MLM: Which part of the body have you treated mostly?

RESP: Only legs and hands and people with dislocation in the hip bone.

MLM: Do you correct people with broken cartilages?

RESP: I just massage, cut the area a little bit and put a medicine

MLM: Do you grind oltemwai before puting to the wound.

RESP: The oltemwai looks like oil so you just rub it on the wound.

MLM: Is the cost of setting legs, arms and cartilages the same?

RESP: It is the same 170,000Tsh

MLM: How did you first learn this treatment?

RESP: I immitated from my father.

MLM: Is there any other relative or brother or sister of yours who also imitated?

RESP: No one else it is only me.

MLM: How many are you in the family?

RESP: we are five children.

MLM: Did the rest try and fail or they had never tried?

RESP: They have never tried

MLM: How long have learned from your father?

RESP: I learned from him for three years.

MLM: Did he teach you or you immitated?

RESP: He teach me as well as I immitated. I was with him during treatment and he ask me do this do that and finally I learn a number of things.

MLM: Does your mother do this treatment?

RESP: No

MLM: Was your grandpa a bone setting specialist?

RESP: Yes he was doing it and that is where my father imitated.

MLM: Have ever received any training about your work?

RESP: I have never received any training.

MLM: What do you recommend if you look at the hospital treatment in bones and the treatment you provide as traditional bone setters?

RESP: They are all very good and they are doing something good. The problem with the hospital, once they had put a patient a POP they do not make followups nor giving any medicine. That is why when a patient who was once set in the hospital fall there is a likely chance that the same place might broke again. For us we make followups.

MLM: Do you still make followups after the patient has fully recovered?

RESP: No I will not make followups

MLM: Do you use or know any of the hospital procedures in your treatment

RESP: No at all.

MLM:Is there any other treatments in bones that you can't do?

RESP: Yes. I cant help people with broken backbones and heads.

MLM: Are you still working with your father?

RESP: There some treatment every body perform individually but he may call me if there is something he can do because of the old age.

MLM: Have you ever referred a patient to the hospital?

RESP: No because I have been receiving arms and legs cases.

MLM: What do you do to those patient you cant treat?

RESP: For those that I can not treat I order his/her relatives to take him/her to the hospital. There was one patient with a broken neck and they brought him to me, I fail to treat and ask them to take the person to hospital.

MLM: Is it possible to for you to be connected to the hospital doctors so that you can refer complicated patients?

RESP: Yes there is no problem it is possible

MLM: Do agree to be taught hospital techniques that you will be using in your work?

RESP: There is no problem about that because the knowledge I have is God's gift, and unity is good because the masai wise word say " One pillar can not support the house"

MLM: What should be done to improve hospital treatment on bones and your own treatment on bones?

RESP: The only thing to say is that it is better we build unity between us and the hospital. If there is a case that the hospital can't do they can consult me and if there is any case that I can't treat then I consult the hospital

MLM: Is there any thing else to tell us?

RESP:. No

MLM: Is there any other side diseases for a patient with broken bones?

RESP: No

MLM: Do you have anesthetic?

RESP: I don't have but for a person who has joint pain and legs pain (we call it tambasi) we give him/her olgilai.

MLM: Is olgilai taken by drinking?

RESP: Yes it is boiled and drinked

MLM: Where do you get olgilai?

RESP: It is found in the bush far away.

MLM: Is there anything we did not ask which you think that it important to ask?

RESP: no

MLM: What do you recomend so that to improve our research?

RESP: I don't have anything to recommend but I request for feedback at the end of the research.

Demographic information removed

TBS 2: Moshi

Interview conducted in English and Swahili, translated and transcribed by ML

MLM: Tunakuhoji kuhusu tiba ya mifupa kwasababu tunaamini ujuzi wako ni wa muhimu kwaajili ya tiba hiyo hasa katika mkoa wa kilimanjaro. We are interviewing you because we understand that the knowldge you have is important for the born setting in kilimanjaro region.

Unaswali lolote la kuutuuliza kabla hatujaendelea? Do you have any question before we proceed?

RESP: Sina swali. No

MLM: Unapoona watafiti wanakufuata kwa mara ya kwanza unapata mawazo gani? What are your perception when you are firstly approached by the researchers?

RESP: Mawazo yangu ni kwamba ni kueleweshana. I understand that you came so that we learn from each other

MLM: Unaweza kutuelezea ni uduma gani unatoa hapa? Can you tell us which services do you provide?

RESP: Hapa tunatoa udumi nyingi. We provide a lot of services

MLM: Enhee. enhee

RESP: Huduma ya chango kwa wanawake, wanawake ambayo hawapati ujauzito, chango kwa wanume, magonjwa ya sinaa, kisukari,macho, kichwa kuuma, kuna ugonjwa inaitwa, kuna ugonjwa inaitwa mawasisiri, UTI. We treat infertility in women and men, sexual transmitted diseases, diabetes, eyes, headache, also another diseasese known as mawasiriand also Urinary Truct Infections UTI

MLM: Unatibu watu waliofunjika? Do you set bones for those broken people?

RESP: Ndiyo tunatibu mtu aliyefunjika mfupa. Tunamfunga kwa kutumia vipande vya mti naa kuna mafuta fulani yanayotoka kwenye mapango fulani ambapo ndo tunaendelea kumpaka isikaze sana ambapo mtu anaendelea na dawa ya kunywa anakunywa kutwa mara tatu, tunatoa huduma hiyo kwa

mwezi alafu tunaendelea kumkagua wenyewe. Yes we treat by tying the patients using pieces of woods and there is a certain oil that is found in certain caves. We apply the oil in top of the woods to stop them tightening the patient body. After that the patient continue taking the medicine three times a day. We provide the service for a month and we continue making followups.

MLM: Mnatibu mgonjwa ambaye mfupa imetoka njee na yule ambaye haijatoka? Do you treat patients where the bones had protruded outside the skin and the one with covered broken part?

RESP: Ndiyo tunatibu wote maana unajua zile mbao zetu ni dawa, sasa ile inarudisha zile mifupa ndani na zile dawa zinazaidia kufanya mfupa iunge vizuri. Tunarudisha zile nyama alafu tunarudishia ule mfupa pale sasa zile mbao ni dawa, ndiyo inaanza kujiuunga na huku mtu akiendelea kunywa dawa ambapo hapo hapo kuna yale mafuta niliokuambia, tupaka ili zile mbao zizikaze wakati inaendelea kupona na lazima ule mfupa zinaungana. Na hizi dawa zetu ndo zinarekebisha kila kitu maana kuna dawa za kukausha zile nyama ambao anakunywa asubuhi, mchana na jioni. Yes we treat, you know the piece of wood them selv are herbs which help the bones to reset. If the bone protrude we return the born inside and tie with the woods. The pieces of woods has the herb that make those bones to connect again. Also we have the oil that I told you. Also the medicine help the wound to dry . the medicine are taken three times a day.

MLM: Huo mti mnaotumia kutengeneza hizo mbao unaitwaje? What is the name of the tree used to make the pieces of woods?

RESP: Unaitwaje mringo, nayo ni dawa. It is called mringo and it is a herb

MLM: Huu mringo inazaidia nini? What does the mringo help?

RESP: Ule unazaidia kuufuta mifupa iliofunjika kuukutanisha na kuungana. It help to pull the broken bones toward each other.

MLM: Huo mringa mnapata wapi? Where do you get the mringo?

RESP: Huo mringa tunapata Tanga. We get it in Tanga.

MLM: Huo mti mnaufuata muda gani? At what time do go and pick the mringo tree?

RESP: Ule mti huwa tunafuata na kama unapoona tuko wawili na kama kuna tukio la mtu kufunjika mmoja wetu anaenda kufuata na kuleta ila kunakuwepo na hakiba ili izaidie kwa ghafula, mara nyingi tunafuata kwa mwezi mara mbili. As you see we are two here. When the patient occur one of us will go and pick the mringa tree. We also make sure that we have extra mringa pieces stored for emergence. Mostly we pick twice a month.

MLM: Kitendo cha kukandakanda kuurudisha mfupa maahala pake inachukua muda gani? The act of massaging the bone into its position takes how long?

RESP: Kukandakanda mfupa na maji, tunakanda mtu kwa siku tatu kabla ya kufunga, na kuna dawa ya kuweka kwenye yale maji ya moto, tunakanda na hayo maji yenye dawa ili mtu asiwe na maumivu wakati anafungwa. Na tunakanda mara mbili kwa siku. Wemassage the bone with worm water for three days before tying. There is a herb that is put in the warm water which help the patient from pain during the tying process.

MLM: Hiyo dawa mnaoweka kwenye maji ya moto inaitwaje? What is the name of that herb that you put in warm water?

RESP: Ni mafuta ya ubuyu. It is an oil from the baobab tree.

MLM: Mnakanda kwa kutumia mikono? Do you massage using hands?

RESP: Ndiyo lakini tunakuwa na gloves zile za kawaida za kununua dukani. Yes but we have normal gloves from the shop.

MLM: Mnatumia nini kuifungia zile pande za mbao? What do yuo use to tie the two pieces to the body?

RESP: Kuna mti mwingine unaitwa mlawa, mti huu inadawa na ndo tunatoa kamba za kufungia zile mbao. There is another tree known as mlawa. This tree has herb and that is where we make wropes that will be used to tie the piece of woods.

MLM: Hiyo dawa ilioko kwenye hizo kamba inazaidiaje? What does the herbs in the wrope help?

RESP: Zile kamba zinadawa ambayo inazaidia kushikisha mbao vizuri kwenye sehemu iliofunjika na wakati wa kufungua tunatumia maji ya moto yenye mafuta ya ubuyu. The herb in the wropes help the piece of woods to hold firmly to the broken part and the time we open we use warm water with oil from the baobab tree.

MLM: Kwahiyo hizo kamba hazina dawa? So that wropes has no herbs?

RESP: Hazina dawa ila inazaidia tu kushikisha zile mbao kwenye sehemu iliofunjika. They had no herb, they just help to hold on the pieces of woods.

MLM: Inachukuwa muda gani kutolea zile mbao? How long does it take to remove the pieces of woods?

RESP: Inategemea na mgonjwa kuna wengine wamefunjika vibaya na wengine kinga yao siyo nzuri sana. Ila tunamzimamia kwa muda wa mwezi na kuanza kumpatia mazoezi na kama akipona tunamuachia aende. It depends on the patient some are terrebly injured and others were injured when their immunity is in truoble so we check the patient for a month and he/she recovers we released

MLM: Kama mtu hajapona baada ya muda huo mnaendelea na zile mbao? If the person did not recover do you continue with the same pices of woods?

RESP: Kama mtu hajapona tunabadilisha kuweka mbao zingine maana zile za mwanzo uenda dawa yake imepungua. If the person did not recovered we change the wood because there is a possibility that the previous one has loose its herbal ability.

MLM:Baada ya mtu kuonekana amepona mnaondoa vitu vyote? Do you remove everything after the recovery?

RESP: Ndiyo tunaondoa vitu vyote na kukaa naye kwa siku sita kuona maendeleo yake. Yes we remove every thing but we retain the patient for six days to see his/her progress.

MLM; Na mtu akiondoka baada ya kupona mnamba dawa ya kuenda kunywa? Do you give the patient herbs to go and use at home after recovery?

RESP: Ndiyo tunampatia dawa. Yes we over some herbs

MLM: Hizo dawa zinaitwaje? What is the name of the herb?

RESP: Kuna mtundwi, muinu, mjazikai, ufunguo na zote zinachanganywa . There is one known as mtundwi, muinu, mjazikai and ufunguo and they are all mixed together.

MLM: Mtu anakunywaje? How does the person drink?

RESP: Mtu anakunywa kijiko kimoja ya mchanganyiko ya dawa hizi kwa siku mara tatu na anaweka kwenye uji au chai lakini siyo maziwa. He/she take one spoon full of the mixture of the herbs three times a day and he mix in porage of tea but not milk.

MLM: Hizi dawa zinazipatikana? Are this herb available?

RESP: Zinapatikana Tanga. They are found in Tanga

MLM: Hizo dawa zinazaidia nini? What does the herb help?

RESP: Hizi dawa zinazaidia kupunguza maumivu na kumfanya mtu kuwa vizuri wakati mtu anapokuwa kwenye baridi inazaidia kulinda mifupa na mazingira. It acts as pain killers but also warm the bones during cold weather.

MLM: Hizi dawa nne zinafanya kazi moja? What does the four herbs do?

RESP: Hizi dawa zinafanya kazi nyingi katika mwili, inazaidia kutibu malaria, kuongeza nguvu za kiume, kama mwanaume hajisikii kufanya tendo la ndoa inazaidia. Na tumpatia mteja wetu kama zawadi. These herbs does a lot in the body, it cure malaria, improve fertility and sometime we give our patients as gifts.

MLM: Wakati wa matibabu hawa watu wanakaa wapi? Where do your patients stay during treatment?

RESP: Nyumba tunayoishi inavyumba kama tatu hivi na wagonjwa wetu wanakaa kwa mwezi chini ya ungalizi wetu. They stay in our house for a month under our observation.

MLM: Mnakaa nao hapo? Do you stay with them there?

RESP: Ndiyo anakaa hapa kwa mwezi mzima. Yes they stay here for a month.

MLM: Endapo mtu hajapona baada ya kumjaribu unaendelea kukaa naye pale? If the patient did not recovered after a month will you go on staying with?

RESP: Ndiyo tunaendelea kukaa naye hapo na kumuudumia. Yes we continue serving him/her.

MLM: Kama imetokea mfupa imetoboa ngozi kwahiyo kuna kidonda mnafanyaje? What will you do if the bone has pearse the skin?

RESP: Tunatibu kidonda na mfupa. We treat the wound as well.

MLM: Sasa mkifunga na zile mbao zi utafunika sasa kidonda? If you tie with wood will you not hert the patients wounds?

RESP: Hapana tunakitambaa kizuri laini na tunaweka dawa na kuweka pale kwenye kidonda kabla hatujafunga. No we have a very clean soft piece of cloth with herb that we put on the wound before tying.

MLM: Hiyo dawa mnayoweka kwenye kitambaa inaitwaje? What is the name of the herb you put in the soft white cloth

RESP: Hiyo dawa inaitwa mrututu. That herb is called mrutu.

MLM: Mrututu inafanya kazi gani? What does mrutu help?

RESP: Inauwa bakiteria nakuzuia kidonda kutooza na kuikausha kidonda. It kills bacteria and prevent the wounds from rotting and dries the wound.

MLM: Hiyo kitamba mnapata wapi? Where do you get the mrutu?

RESP: Tunanunua special kwaajili ya kazi hiyo. Tunachukua kitambaa cheupe dukani na pamba kwaajili ya kuweka dawa kama vile daktari anavyofunga kidonda. We buy it specifically for this work. We buy the soft white cloth and the cotton wool so as to use to apply the herb on the wound.

MLM: Huwa mnabadilisha kitambaa? Do you always change the piece of cloth?

RESP: Ndiyo tunabadilisha kitambaa. Na zile mti tunazibadilisha pia. Yes we change the wood and the piece of cloth.

MLM: Mara nyingi mnabadilisha baada ya muda gani? After what time do you change the woods and the piece of cloth?

RESP: Baada ya siku mbili na tunasafisha na maji ya moto. Afater two days and we clean using warm water.

MLM: Hivyo vipande vya mti vinaukubwa gani? How large is the pieces of woods?

RESP: Kama mbao hii (anaonyesha mfano wa mbao) ila nyembamba zaidi. Like this wood (showing a certain wood there) but thiner than this.

MLM: Unaweza kumtibu mtu ambaye alishafunjika akaungwa akapona lakini bado mguu au mkono umepinda? Will you treat a person who was once treated or set wrongly leading to the arm or leg to become lamed?

RESP: Ndiyo tunamtibu mtu huyu, hatuvunji mguu tena ila tunafivyaa vyetu ambazo zinamzaidia ule mtu. Yes we treat the person. We do not broke but we do somethings to help the person

MLM: Hivyo fivyaa venu ni zipi? What are those things?

RESP: Sisi kuna mti wetu unaitwa mvumo na huo mti inatoa ute mweupe kama karatazi na tunapa juu ya mtu aliyefunjika akaungwa vibaya kwenye eneo lililofunjikaga, baada ya kuweka hiyo ute hapo tunafunga bandage kama POP inapofungwa. We have a certain tree known as mvumo, it has a white gluish content. This content is smeared above the bandage and act like a POP and the person bones region correctly.

MLM: Baada ya hapo mnasubiri kuona kama atapona? So after that you wait to see if he recovered?

RESP: Ndiyo baada ya wiki mbili na kuifungua kuona kama amepona ama anasikia maumivu. Na kama mtu amepona tunamuachia lakini kama amepona anaondoka. Yes after two weeks we remove the POP and when has recovered we released him.

MLM: Mvumo nayo inakuwa ngumu kama POP? Is the mvumo hard like a normal POP?

RESP: Ndiyo inakuwa kama POP na ukiwekewa hapa uwezi kutofautisha labda kwa mtu anaelewa. Yes it just like a POP and if you put it here you can not compare may be for a specialist like me.

MLM: Mvumo inapatikana wapi? Where do you get the mvumo?

RESP: Inapatikana sehemu inaitwa msaranga. It is obtain in a place known as msaranga

MLM: Ni wewe ndo unaenda kuchukuwa? Are you the one who go for it?

RESP: Ni mimi hapa ndo naenda kuchukuwa. Yes I go for it.

MLM: Inapatikana? Is it easly available?

RESP: Ndiyo inapatikana. Yes it is available.

MLM: Unatibuje mtu aliyefunjika muda mrefu mnamkanda pia? Do you treat long time injuries and do you massage as well?

RESP: Hatumkandi huyo. Yes but we don't massaged.

MLM: Wewe umekuwa ukitibu sehemu gani za mwili? Which parts of the body are common in your treatment?

RESP: Miguu, mkono au mishipa ikipishana, tunatumia blangeti nzito na kuweka dawa kwenye blanketi na kuizungusha mgongoni kama mishipa imepishana mgongoni. Legs, hands, mislocations in bllod nerves. We use a big blanket and put a herb on a blanket and wrap it around the back if the nerves seem to dislocate in the backbone.

MLM: Hiyo dawa inaitwaje? What is the name of the herb put in a blanket?

RESP: Inaitwa msono? It si called msono.

MLM: Je kama mtu amefunjika unyayo , mgongo, shingo mnatibu? Do you treat bones broken in the pulm, back bone and the neck?

RESP: Ndiyo tunafanya kote ila mara nyingi ni mifupa ya miguu. We do all but mostly on the legs bones.

MLM: Huwa mnakata ngozi wakati wa kutibu? Do you make mark or cut the skin during treatment?

RESP: Ndiyo tunakata endapo kuna huitaji wa kukata na kama kuna nyama zimechanika vibaya na kuzuia mfupa ikaye vizuri na tuna mikazi yetu. Yes we cut when necesary or when there is a piece of meat that prevents the bones to set correctly. We have our own scissors.

MLM: Je mnachanja sehemu iliofunjika ili kuruhusu dawa iingie? Do you make a small mark on the broken area to allow the medicine to enter?

RESP: Hatuchanji . No

MLM: Je unatoa tiba yeyote ya kiimani? Are a religious healler?

RESP: Hapana sisi hatuangui ni tiba ya asili tu labda kama mtu anahitaji maombi tunatafuta mtu wa kumuombea. No we just provide herbs but if a patient needs prayers we find some one to pray for him.

MLM: Je mnawambia wagonjwa wakatambike? Do you tell your patients to make sacrifices?

RESP: Hapana maana huo ni ushirikina. No because that is whichcraft.

MLM: Ni wagonjwa wangapi mnapata wa mifupa kwa mwaka? How many patients do you recieve per year for bone setting?

RESP: Ni kama watu wawili kwa mwaka. Toka julai mwaka jana hadi julai mwaka huu tumetibu watu wawili waliofunjika miguu kutokana na ajali. About two people per year. Since july last year up to july this year we have received two patients from the road accident.

MLM: Kazi hii ya tiba ya mifupa ndo chanzo ya kipato chako? Is this work the source of your income?

RESP: Ndiyo lakini nakuwa na kazi hii ya kupeana dawa na hii ya kupeana dawa ndo inayonilipa zaidia zaidi kwasababu hii ya kufunjika mtu inatokea mara chache. Yes but I also administer the herbs and it is the main source of income

MLM: Huwa mnadai mtu shilingi ngapi unapomtibu kwa wale waliofunjika mifupa? How much do you charge one patient when setting bones?

RESP: Tunamdai mtu laki tatu lakini mtu anatoa laki na nusu mwanzoni na kumalizia atakapopona. We charge 300,000Tshs but he/she pay an advance of 150,000Tshs then the rest after recovering.

MLM: Kama mtu hajapona mkamrudia tena mtadai gharama nyingine? If the person did not recover and you repeat the treatment will charge him/her again?

RESP: Hapana gharama ileile. No we do not charge again.

MLM: Wewe umejifunza wapi hii tiba? Where did you learn this treatment?

RESP: Nimejifunza kwa babu yetu ila pia tumekuwa tukienda semina na mafunzo mbalimbali. I learn from my father but I have attended several seminars on this treatment.

MLM: Semina mlienda wapi? Where was the seminar?

RESP: Tulienda tanga, arusha, moshi hapahapa. We attended on in Tanga, Arusha and one in moshi.

MLM: Unajua nani alikuwa anaendesha hiyo semina? Do you know who was facilitating the seminar?

RESP: Labda kwa kuangalia yale makaratazi ndo nitajua ni kina nani. May be until I re-visit the document is when i will who was facilitating.

MLM: Je mnafundishwa na madakitari wa kiasili kama nyie? Were you tought by traditional specialist like you?

RESP: Hapana tulifundishwa na maafisa utamaduni na hao watu wanatumwa na serikali na ndo wanaoturuhusu kufanya kazi hii. No we were tought by the cultural officers from the Government and they are the one to authorized us to do this treatment.

MLM: Hii ujuzi imetoka kwa babu kuja kwa baba au na je kuna watu wengine wamejifunza? Was this knowledge inherited from the grand father to the father then to you, and are there other family members who learned this?

RESP: Hapana imetoka kwa babu kuja kwangu maana mimi ndo nimechukuwa jina lake, na mimi kama kuna mtu atakaye taka nitamfundisha. No it was from the grand father to me because I am the one who inherited his name. And if there is any one who will need to know I will teach him.

MLM: Kuna kijana wako anajifunza? Is there one of your sons who is learning?

RESP: Ndiyo kuna kijana wangu anajifunza. Yes there is my son who is learning.

MLM: Hakuna msichana anayejifunza? Is there a daughter who is learaning?

RESP: Hakuna msichana anayejifunza. No girls

MLM: Wewe na shebuke ni ndugu na mnashirikiana kwenye kiliniki hii? Are you related to Shebuke and are you sharing the clinics?

RESP: Ndiyo tunashirikiana na sisi ni ndugu. Yes we are working together and we are relatives.

MLM: Je kuna watu wengine mnashirikiana nao kwenye kiliniki hii? Are there any other subodinate in your clinic that your working together?

RESP: Wapo watu wengi wa tiba ya asili lakini kila mtu kuna jambo analoweza zaidi. Wengine wanatibu macho, meno, wengine magonjwa mbalimbali. Yes there are other specialist but with different skills, some treat eyes, teeth and other diseases.

MLM: Je uwezo wenu ninyi ni nini? What is your skill?

RESP: Sisi tunatibu mifupa na magonjwa meengine mbalimbali. We set bones and treat other various diseases

MLM: Unafikiriaje kuhusu uduma ya mifupa ya hosipitali? How do you consider the hospital bone setting?

RESP: Ya hosipitali ni nzuri na yetu pia ni nzuri lakini mimi ningependelea hii yetu maana tunajiamini kuhusu uwezo wetu kwenye tiba hii. The hospital bone setting is good and ours is also good but I prefer mostly ours because we have courage on bone setting.

MLM: Je mnatumia utaratibu za kihosipitali wakati mnafanya tiba hii? Do you use hospital procedures when doing this treatment?

RESP: Ndiyo tunakuwa na kibali ya kufanya tiba. Yes we are authorized to do this treatment

MLM: Kibali mnapata wapi? Where do you get the permit?

RESP: Tunapata manispaa. From the municipal governemnt.

MLM: Ni vitu gani vya hospitali mnatumia kwenye tiba yenu? Which hospital equipments do you use in your treatment?

RESP: Tunakuwa na gloves, bandage, mikazi na sufuria za kuchemshia mbao na mikazi zetu. We use bandages, gloves, scissors and boil pots for sharp tools.

MLM: Je mnashona wagonjwa? Do you sitch patients?

RESP: Hapana. No

MLM: Je mna ex-rays? Do you have ex-rays machine?

RESP: Ex-ray hatuna. No we don't have ex-ray machine.

MLM: Je mnayomadawa kama antibiotics na ya maumivu? Do you have antibiotics and pain killers?

RESP: Ndiyo lakini ni hizi za asili siyo ya hospitali. Yes but not the hospital one it is the traditional one.

MLM: Mnapata wapi gloves? Where do you get the gloves?

RESP: Tunanunua kwenye maduka ya dawa. We buy them from pharmacy

MLM: Je kuna matatizo ya mifupa ambayo hamuwezi kutibu? Are there bone problems that you can't treat?

RESP: Labda kama imekuwa checked, au kama mifupa imezagika sana au kama mtu amepasuka kichwa hadi kwenye ubongo. Yes when the person has been already checked, when the bones fractures are to severe or when the skull is terrebily injured.

MLM: Je unaweza kumuagiza mtu kwenda hospitali au kwa dakitari wa kiasili kama wewe? Do you refer the patients to the hospital or to the other specialist like you?

RESP: Ndiyo kama siwezi kutibu namuagiza hospitali ya KCMC na Mawenzi na nilishawahi kumuagiza mtu aende kwa mtaalamu mwingine wa mifupa aliyoko Morogoro. Yes if I can't treat i refer them to KCMC or to Mawenzi and I had one case that I refered to another bone specialist in morogoro.

MLM: Kwanini ulimuagiza huyo mtu morogoro? Why did you refer the patient to that specialist in morogoro?

RESP: Unajua unaweza kumuangalia mtu unaona umuwezi unaongea na mtaalamu mwenzako unampeleka au unamuagiza. Sometime after diagnosing a patient and new that his/her problemis beyond your abilities you call another specialist and explain the situation to him. You can refer or you can take the patient your self.

MLM: Je utakuwa tayari kuwa na ushirikiano na muunganiko na wataalamu wa hospitali katika tiba hii? Will be ready to work with other specialist in the hospital who are rsponsible in bone setting?

RESP: Ndiyo kwa kuwa tutashirikiana kimawazo katika kazi hii. Yes because we will share ideas about this work.

MLM: Kwa mfano ikitokea, utakubali kujifunza mbinu zingine za kihospitali katika tiba hii? For example, when it happens, are you ready to be trained on the hospital procedures of doing this work?

RESP: Ndiyo nitakubali. Yes I will agree

MLM: Unaweza kushauri chochote kwwajili ya kuongeza tija katika tiba hii hospitalini na kiasili? Can you advice anything in improving the bone setting both in hospital and traditional ways?

RESP: Ndiyo cha muhimu ni kuja pamoja na kushirikiana katika kufanya kazi hii na kuona mapunguvu yaliopo kwenye tiba hii ya asili na yale yaliopo kwenye tiba za kihospitali. Yes. The best way is coming together, working together and identifying shortages in hospital procedures and in the traditional procedures.

MLM: Wewe unaionaje huduma hii yako katika jamii au unaelezeaje mchango wako katika jamii? How dou recoment your treatment or your contribution to the community?

RESP: Mchango wangu ni nzuri na inatambuliwa na wateja shida tu ni kwamba sina uwezo wa kuanzisha kiliki ya kisasa. My contribution has been perfect and accepted widely by the community. The only problem is that I am unable to start a modern herbal clinic.

MLM: Je kunakitu kingine unaweza kutuambia ambacho ujaweza kusema? Do you have anything that you can tell us more?

RESP: Hakuna kwakweli. No for sure.

MLM: Je kuna kitu unaweza kutushauri kufanya ili kuboresha mahojiano yetu? Is there anything you can advice so that we improve our interview?

RESP: Sina ila kama kuna kitu hamja uliza ulizeni tu. I don't have and if you still have questions fill free to ask.

MLM: Umekuwa ukifanya hii kazi kwa muda gani? How long have been working as bone setter?

RESP: Nimekuwa nikifanya kazi hii kwa muda wa miaka ishirini na nne. For about twenty four years

AHSANTE SANA. THANK YOU

Demographic information removed

TBS 2: Moshi, ENGLISH ONLY TRANSCRIPTION

Interview conducted in English and Swahili, Swahili translated/transcribed by ML, additional English transcription by EBC

MLM: We are interviewing you because we understand that the knowledge you have is important for the born setting in kilimanjaro region. Do you have any question before we proceed?

RESP: No

MLM: What are your perception when you are firstly approached by the researchers?

RESP: I understand that you came so that we learn from each other

MLM: Can you tell us which services do you provide?

RESP: We provide a lot of services

MLM: Enhee.

RESP: We treat infertility in women and men, sexual transmitted diseases, diabetes, eyes, headache, also another disease known as mawasiri and also Urinary Tract Infections UTI

MLM: Do you set bones for those broken people?

RESP: Yes we treat by tying the patients using pieces of woods and there is a certain oil that is found in certain cafes. We apply the oil in top of the woods to stop them tightening the patient body. After that the patient continue taking the medicine three times a day. We provide the service for a month and we continue making followups.

MLM: Do you treat patients where the bones had protruded outside the skin and the one with covered broken part?

RESP: Yes we treat, you know the piece of wood are herbs which help the bones to reset. If the bone protrude we return the born inside and tie with the woods. The pieces of woods has the herb that make those bones to connect again. Also we have the oil that I told you. Also the medicine help the wound to dry. the medicine are taken three times a day.

MLM: What is the name of the tree used to make the pieces of woods?

RESP: It is called mringo and it is a herb

MLM: What does the mringo help?

RESP: It help to pull the broken bones toward each other.

MLM: Where do you get the mringo?

RESP: We get it in Tanga.

MLM: At what time do go and pick the mringo tree?

RESP: As you see we are two here. When the patient occur one of us will go and pick the mringa tree. We also make sure that we have extra mringa pieces stored for emergence. Mostly we pick twice a month.

MLM: The act of massaging the bone into its position takes how long?

RESP: We massage the bone with warm water for three days before tying. There is a herb that is put in the warm water which help the patient from pain during the tying process.

MLM: What is the name of that herb that you put in warm water?

RESP: It is an oil from the baobab tree.

MLM: Do you massage using hands?

RESP: Yes but we have normal gloves from the shop.

MLM: What do you use to tie the two pieces to the body?

RESP: This tree has herb and that is where we make ropes that will be used to tie the piece of woods.

MLM: What does the herbs in the rope help?

RESP: The herb in the ropes help the piece of woods to hold firmly to the broken part and the time we open we use warm water with oil from the baobab tree.

MLM: So that rope has no herbs?

RESP: They had no herb, they just help to hold on the pieces of woods.

MLM: How long does it take to remove the pieces of woods?

RESP: It depends on the patient some are terribly injured and others were injured when their immunity is in trouble so we check the patient for a month and he/she recovers we released

MLM: If the person did not recover do you continue with the same pieces of woods?

RESP: If the person did not recovered we change the wood because there is a possibility that the previous one has loose its herbal ability.

MLM: Do you remove everything after the recovery?

RESP: Yes we remove everything but we retain the patient for six days to see his/her progress.

MLM; Do you give the patient herbs to go and use at home after recovery?

RESP: Yes we over some herbs

MLM: What is the name of the herb?

RESP: There is one known as mtundwi, muinu, mjazikai and ufunguo and they are all mixed together.

MLM: How does the person drink?

RESP: He/she take one spoon full of the mixture of the herbs three times a day and he mix in porage of tea but not milk.

MLM: Are this herb available?

RESP: They are found in Tanga

MLM: What does the herb help?

RESP: It acts as pain killers but also warm the bones during cold weather.

MLM: What does the four herbs do?

RESP: These herbs does a lot in the body, it cure malaria, improve fertility and sometime we give our patients as gifts.

MLM: Where do your patients stay during treatment?

RESP: They stay in our house for a month under our observation.

MLM: Do you stay with them there?

RESP: Yes they stay here for a month.

MLM: If the patient did not recovered after a month will you go on staying with?

RESP: Yes we continue serving him/her.

MLM: What will you do if the bone has pierce the skin?

RESP: We treat the wound as well.

MLM: If you tie with wood will you not hurt the patients wounds?

RESP: No we have a very clean soft piece of cloth with herb that we put on the wound before tying.

MLM:What is the name of the herb you put in the soft white cloth

RESP: That herb is called mrutu.

MLM: What does mrutu help?

RESP: It kills bacteria and prevent the wounds from rotting and dries the wound.

MLM: Where do you get the mrutu?

RESP: We buy it specifically for this work. We buy the soft white cloth and the cotton wool so as to use to apply the herb on the wound.

MLM: Do you always change the piece of cloth?

RESP: Yes we change the wood and the piece of cloth.

MLM: After what time do you change the woods and the piece of cloth?

RESP: After two days and we clean using warm water.

MLM: How large is the pieces of woods?

RESP: Like this wood (showing a certain wood there) but thinner than this.

MLM: Will you treat a person who was once treated or set wrongly leading to the arm or leg to become lamed?

RESP: Yes we treat the person. We do not break but we do somethings to help the person

MLM: What are those things?

RESP: We have a certain tree known as mvumo, it has a white gluish content. This content is smeared above the bandage and act like a POP and the person bones region correctly.

MLM: So after that you wait to see if he recovered?

RESP: Yes after two weeks we remove the POP and when has recovered we release him.

MLM: Is the mvumo hard like a normal POP?

RESP: Yes it just like a POP and if you put it here you can not compare may be for a specialist like me.

MLM: Where do you get the mvumo?

RESP: It is obtain in a place known as msaranga

MLM: Are you the one who go for it?

RESP: Yes I go for it.

MLM: Is it easily available?

RESP: Yes it is available.

MLM: Do you treat long time injuries and do you massage as well?

RESP: Yes but we don't massaged.

MLM: Which parts of the body are common in your treatment?

RESP: Legs, hands, mislocations in blood nerves. We use a big blanket and put a herb on a blanket and wrap it around the back if the nerves seem to dislocate in the backbone.

MLM: What is the name of the herb put in a blanket?

RESP: It is called msono.

MLM: Do you treat bones broken in the foot, back bone and the neck?

RESP: We do all but mostly on the legs bones.

MLM: Do you make mark or cut the skin during treatment?

RESP: Yes we cut when necessary or when there is a piece of meat that prevents the bones to set correctly. We have our own scissors.

MLM: Do you make a small mark on the broken area to allow the medicine to enter?

RESP: No

MLM: Are you religious healers?

RESP:No we just provide herbs but if a patient needs prayers we find someone to pray for him.

MLM: Do you tell your patients to make sacrifices?

RESP: No because that is witchcraft.

MLM: How many patients do you receive per year for bone setting?

RESP: About two people per year. Since july last year up to july this year we have received two patients from the road accident.

MLM: Is this work the source of your income?

RESP: Yes but I also administer the herbs and it is the main source of income

MLM: How much do you charge one patient when setting bones?

RESP: We charge 300,000Tshs but he/she pay an advance of 150,000Tshs then the rest after recovering.

MLM: If the person did not recover and you repeat the treatment will charge him/her again?

RESP: No we do not charge again.

MLM: Where did you learn this treatment?

RESP: I learn from my father but I have attended several seminars on this treatment.

MLM: Where was the seminar?

RESP: We attended on in Tanga, Arusha and one in moshi.

MLM: Do you know who was facilitating the seminar?

RESP: Maybe until I re-visit the document is when i will who was facilitating.

MLM: Were you taught by traditional specialist like you?

RESP: No we were taught by the cultural officers from the Government and they are the one to authorized us to do this treatment.

MLM: Was this knowledge inherited from the grand father to the father then to you, and are there other family members who learned this?

RESP: No it was from the grand father to me because I am the one who inherited his name. And if there is anyone who will need to know I will teach him.

MLM: Is there one of your sons who is learning?

RESP: Yes there is my son who is learning.

MLM: Is there a daughter who is learning?

RESP: No girls

MLM: Are you related to Shebuke and are you sharing the clinics?

RESP: Yes we are working together and we are relatives.

MLM: Are there any other subordinate in your clinic that you're working together?

RESP: Yes there are other specialist but with different skills, some treat eyes, teeth and other diseases.

MLM: What is your skill?

RESP: We set bones and treat other various diseases

MLM: How do you consider the hospital bone setting?

RESP: The hospital bone setting is good and ours is also good but I prefer mostly ours because we have courage on bone setting.

MLM: Do you use hospital procedures when doing this treatment?

RESP: Yes we are authorized to do this treatment

MLM: Where do you get the permit?

RESP: From the municipal government.

MLM: Which hospital equipments do you use in your treatment?

RESP: We use bandages, gloves, scissors and boil pots for sharp tools.

MLM: Do you sitch patients?

RESP: No

MLM: Do you have x-rays machine?

RESP: No we don't have x-ray machine.

MLM: Do you have antibiotics and painkillers?

RESP: Yes but not the hospital one it is the traditional one.

MLM: Where do you get the gloves?

RESP: We buy them from pharmacy

MLM: Are there bone problems that you can't treat?

RESP: Yes when the person has been already checked, when the bones fractures are to severe or when the skull is terrebily injured.

MLM: Do you refer the patients to the hospital or to the other specialist like you?

RESP: Yes if I can't treat i refer them to KCMC or to Mawenzi and I had one case that I referred to another bone specialist in Morogoro.

MLM: Why did you refer the patient to that specialist in Morogoro?

RESP: Sometime after diagnosing a patient and new that his/her problem is beyond your abilities you call another specialist and explain the situation to him. You can refer or you can take the patient yourself.

MLM: Will be ready to work with other specialist in the hospital who are responsible in bone setting?

RESP: Yes because we will share ideas about this work.

MLM: For example, when it happens, are you ready to be trained on the hospital procedures of doing this work?

RESP: Yes I will agree

MLM: Can you advice anything in improving the bone setting both in hospital and traditional ways?

RESP: Yes. The best way is coming together, working together and identifying shortages in hospital procedures and in the traditional procedures.

MLM: How do you recommend your treatment or your contribution to the community?

RESP: My contribution has been perfect and accepted widely by the community. The only problem is that I am unable to start a modern herbal clinic.

MLM: Do you have anything that you can tell us more?

RESP: No for sure.

MLM: Is there anything you can advise so that we improve our interview?

RESP: I don't have and if you still have questions feel free to ask.

MLM: How long have been working as bone setter?

RESP: For about twenty four years

AHSANTE SANA. THANK YOU

Demographic information removed

TBS 3: Arusha

Interview conducted in English and Maa, translated/transcribed by ML.

MLM: Ejoito toi aikioto duo pee kingilikwanishore iyie amu kidol duo aajo ore eng'en niata oo enaimaki iltung'an, naa eng'eno nagut oleng naretokito iltung'anak oogila neeku ninye pee kietuo akilikwanishore iyie. We are interviewing you because the knowledge you have on bone setting is important in helping people with brokens bones.

RESP: Esipa. True

MLM: Kamaa eton itu kipuo dukuya, iata oswali lingilikwan iyiok? Do you have any question before we proceed?

RESP: Maata amu ameikimboto ninye pee kiponunu aining, kake anyor sii tukul iyetuo. I dont have because you have called me so that you listen from me but i really appreciate this.

MLM: Kanyoo engibiribirata ino edukuya enidol ilaikeernok kiponu aikidikwanishore? What are your first thoughts when first approached by researchers?

Anyor tukul amu metaana oshi ake aing'oru, aibung'a apa ang'amaa ilaigwanak, aishoo ninye olbungei embalani, nelotu olengarioi neya impala manga, nelotu olekakanyi naajoki tisiru lelo tung'anak kumok litabaa, nasir neya mayelo kaakeshomo aiminie, mayelo. I reallylike because i have been apprached by community leaders trying to document all that i do. I gave them information.

MLM: Kakwa ramatat duo oshi injo iltung'anak? Which services do you over to your patients?

RESP: Maa enjoni oshi iyiok kipik iltung'anak, akashor ake oltung'ani engaina, nashor tukul aitorioo, ee engeju, ee engaina akashor tukul naiteraki olkodoro pee medot enjoni oldung'ani amu enjoni nanu aidikie oltung'ani otigile. I always use hides in setting bones. I massage the broken arm and after that, i tied it with a piece of mattres then the hide. The piece of matress prevent the skin from condacting the hide which might cause bruises.

MLM: Kenjoni nagol toi? Is it hard hide?

RESP: Aaa enjoni ake duo engiteng naa kaen ake inewei kake akaud inashoni naa kaitutu napik inaudot kumok, ore pee esar enjan naitoki aitirish arashu tenelo pee eteja engaina oleng naitololong. Is just a normal dry skin of a cow, i just make several holes on it and change after some time or if the arm swollen i released the skin.

MLM: Iramat kulie moyiaritin enake iloik ake indikidik? Do you treat other diseases or you are just a bone setter.

RESP: Aaa tukul pookin engai toki naas aitaki ingishu ilosho eneye tengoshoke arashu enelaikino atoisho.I do the other thing that I do is helping animals during calving.

MLM: Indikidik oshi ninje ingishu enegila? Also i set bones in animals.

RESP: Ee aidikidik kake akamishir ake tolmishire. I just massage using hot iron.

MLM: Iramat oshi iltung'anak otigilate temusano neeku maiman ishiutuo? Do you treat long injuries that make people lame?

RESP: Ipaaye aramat, kumok iltung'anak oyawaki sipitali nepwei adikidik neriwei ingejek nayakini nagil naitorioo. Yes i do. There many people taken to hospitals and after they recover they become lame. There relatives brought them to me and I broke them and reset them a fresh.

MLM: Iramaat taata duo iltung'anak oya irubat ashu ooning'ito irubat ajo eme? Do you always treat people with joints pains?

RESP: Ipaye eya nayakini oltung'ani oning'ito erubata eme, ore pee ayakini naa kashor aitorioo. Emidamu toi apa engai ayoni engang inyi alaishoni oyewaki isipitalini nelaikinoi pi naa engunja apa nayiwang'a nelauni pii. Nayakini ake nalotu ashor nishiu pii. When they brought to me people of this kind I massage them correct the pain. Do you remember a young boy from your family who belongs to alaishoni? MLM: Yes. RESP: The boy was taken to various hospitals and no success and when they brought to me I treated and he recovered fully.

MLM: Iramat toi iltung'anak ooya ingejek? Arashu oya irubat? Do you treat leg or joint pains?

RESP: Aaaa ore naleng naa lelo otigile. Mostly I teat those with broken bones.

MLM: Kanu apa interua adikidik iltung'anak? When did you started bone setting.

RESP: Musana naleng kira ilmuran alo ilarin osom oima. Long time ago when I am a moran about thirty years ago.

MLM: Kang'ai apa nikinteng'ena enasiai? Who teach you about this treatment?

RESP: Enkai ake amu alpayian apa loonterito odikisho aibung'a aaduaki oomatyiolo sinanu neeku adikidik iltung'anak etamorua ninye. Just God because there was an old man whom I was helping while setting bones and that where I learned to do this work at the time he become too old.

MLM: Kamaa ilo payian apa litaduaka kai ibaikinono? Were you related to the old man?

RESP: Meeta aikishula ake tengang, kake itu toi autaki naleng akataduak ake tenebo oo engariayiano ai. We were not related we are just neiighbours. He did not teach me I just immitated from him.

MLM: Kamaa apa iduakitata ilo payian irara kumok? Were you many while observing the process from that old man?

RESP: Kira kumok kake metii ilotishilaitie, ore ake pee elotu ilo tasat aitianya neeku egila ake oltung'ani nayakini, neeku egila ake oltung'ani nayakini omatayiolo pii. Yes we were many but I was the only one who took into consideration. When the old man died people started bringing the patients to me.

MLM: Kengolong'i aja itaduaka ele payian eramatisho? How many days did you observed the old man before you started doing it your self?

RESP: Iltung'anak okuni ake ataduaka ele payian eramatita. I only observe three cases.

MLM: Itu akata kinteng'en ele payian? Did the old man trained you?

RESP: Itu tukul aiteng'en akataduaka ake, neeku akejo iltun g'anak mayaki oltung'ani otigile ilo tung'ani amu arria ake amu etaduaka apa. Netii tataoi ilayiok lenyenak larisiore kake itu eshilaa. He has never trained me I just immitated from him. I just started doing it after I observed.

MLM: Kaja apa iba pee interu ena ramatare? How old are you when you stated bone setting?

RESP: Aata ake ilarin osom olpisiai. I was about thirty years.

MLM: Ketiai apa interua adikidik oltung'ani? Where did you started bone setting?

RESP: Tenewei ake aterua, naa ore ilo laiterua naa ketonita ake ilo payian amu ninye eyakaki ele tamweiai kake etamorua neeku meekure idim adikidiko naajoki madikidiko eing'orita ore pee aidip naajoki inotoko tukul ore aiteru inakata neeku akaidikidik iltung'anak. Ore apa eenaiterunye naa tenewei ake aramatisho kake etaa oshi ayai ingadoru. I just started here with a patient brought to that old man who by the time was too old to perfom the treatment. He told me to joint the bone under his observation and when had completed he recomented that I did it perfectly. From there I started setting bones.

MLM: Kele kazi toi oshi integemea enake iata kulie siatin? Do you always depend on this work or you have other activities for your income?

RESP: Aaa aata ingishu, alakita taatoi amu ore oltung'ani kitok naa engashe, ore engerai naa embalelo. Kake meninye sii aing'orita amu maata duo iltung'anak laitegemea wuade neeku aata si ingishu. No I

have cows. It is also paying because an elder person pays a cow and a child pays a goat or sheep. But I don't fully depend on it because the patient are no readly available they come occationally.

MLM: Keltung'anak aja iramat talari? How many patient do you treat per year?

RESP: Mayelo taduo amu kumok pii, etii oshi engerai natalaa tekunolong'i, netii olmurani otigile neyauni netii endasat, netii olmurrani lengang olokutaki oyawuaki tedarislam etigila olbodaboda nalotu agil. Elaa eya ake nebaya iltung'ank tomon neilep talari. I don't really remember, there was a child who just released few days ago, there was also a moran and one woman. It is almost ten people in year.

MLM: Kekiyai toi ingadoru enake tenewoi ake iyasisho? Do go to far place to treat people?

RESP: Tenewi ake asisho kake ashom napari manyara nalo aidikidik iltung'anak aare, naidikidiko napari oltung'ani oyeuo aitobir olmutandao tenakop nagil nadikidik. Ilo tung'ani apa laa ninye apa alarria, nelotu engaji entereko nejoki ntereko etii oltung'ani oyiolo aatigila enaina ino ninjiu. Nejo emboto maatigila, najoki elaa magil nanu nejo agili ake, nejo eyautu ingeenda, nagil naidikidik nishiu pii. Kake mee naleng duo oshi alo kulie kwapi. I just work in this area. I went to manyara and from there I came a cross to a lady with bones problems and I treated her. There was one person who was an airtel tower enginear and he had a lame hand. Some people told him about me and ordered that I should meet him. I discouraged him that broking the hand is dangerous perhaps. He said he is ready for anything. I broke the hand and reset again.

MLM: Kamaa enekiakini oltung'ani otigile kai ingo eniramat? How do you set bones?

RESP: Meeta atua naya akaramat ake nanu inoolong, neeku akalo ake aleen. Eneyauni oltung'ani nang'amu aing'uraa openy. Naa pookin ilkeek laisho naa iloodwa, olmisigiyioi, neishoru eilata enger, kake maitoki aisho kule. When the patient is brought to me, I diagnos and set the bone. I give the person iloodwa, olmisigiyioi, oil from a sheep but I he will not be given milk any more.

MLM: Kamaa ena shorota nishor oltung'ani kengata nabaa eya? How long do you massage the patient.

RESP: Meya engata naado naleng, eneya engata naado naleng naa nusu saa ake. It only take like half an hour.

MLM: Kama toi ena shoni kaintaman engeju enake aidung'u intokitin are nien toonjot pokira? Do wrap the hide around the leg or hand or you cut two pieces and tie to the legs and arm?

RESP: Akaitaman engeju naiper tenjoto nabo enashoni, naud tenjoto nabo pee atum ayiemie nena keenda pee ejei engeju naitololong, arashu tenesar engeju netirish aigil. One side of the hide has holes made on it and the other side is spilited so that each split get into a hole and when the leg is swollen i adjusted the hide.

MLM: Kebaa engata naton oltung'ani meyeuni neigut? How long does the patient remain imobile?

RESP: Ore oltung'ani otigile engeju neya nebik olapa tenemutu etigile ainyalaki. Kake ore olitu inyalakino engilata naa iwikii are ake eton. Kake ore olengaina neipang ake. For severe inured person he can stay for about a month. If not severely injured he can only remain for two weeks. For the patient with hands problem he /she can still walk.

MLM: Keyie toi otem oltung'ani enake ishiuo? Are you the one to prove whether the patient has recovered?

RESP: Eee nanu ake olo atem nadol ajo ishiuo najoki meshomo. I just test hiim and when he showed that he has recovered I released him.

MLM: Kengolong'i aja oshi ijur pee iyieleu ajo ishiuo? How many days do test the patient to prove his recovery?

RESP: Eya ake naboitare ake ingolong'i uni, hadi ongwan, ore ninye eton emwei naa nanu ake osujaki pee enejei engeju naleng naa nanu ake oitupuk. Ore kenya ake pee esihiuu naa nanu olak. I may stay with him for three to two days . when he is still sick I make sure that I am near to make sure that i rectify any abnormal occurance. When the person recover I untie the broken part.

MLM: Kanyoo oshi eretoki enailata naishori? What does the sheep oil help?

RESP: Aininye oshi olnjani laamaasai naa keretoki iltamweia. Naa ninye iteri aisho. The sheep oil is the first considered maasai medicine and it the first to be administered to the patient.

MLM: Kamaa kulo keek oshori altamweiai kanyoo eretoki? What does the herbs help the patient?

RESP: Ore kulo keek pokira naa keretoki ele baye metoyio tukul. Kake ore sii ilodua naa olnjani labaye. These two herbs dry up the wound and prevent swelling of the broken part. But also iloodwa is herb for gonorhear

MLM: Kai oshi ingongo eniantaanta? How do you prepare the herb?

RESP: Aikiyeru ele misigiyoi nepiki elita neeku akawok enaa engare. Olkiperari lanjani oshi ake edanyuni neyeri ometodoro ina are neeku ninye eok aitaa engare. Ore iloodwa naa akedong'i ake neyeruni nesili neyerieki euji neishori ele tamweiai. I just boil the olmisigiyioi (piece of a dry stem) to make juice from it and the patient drink it like water. For iloodwa it isgrinded boil seived and made a porage from the juice and is given the patient.

MLM: Kanyoo toi pee meok kule? Why do you forbid the patient from taking milk?

RESP: Akitoki aiteja naleng naa neija ake sininje etiu imotori. It swellen the broken part like wise soups.

MLM: Kamaa eninepu oltung'ani otuudo oloito enjoni kaja ingo? How do you treat a patient with a protruding bone above the skin?

RESP: Aiwore enaiko naa tenaen eletung'ani otigile naa akaud enashoni naud enakodoro pee meibookino olpile netumi sii atipik ilkeek. I just make a hole on the hide and the piece of matress equal to that made by the bone so that to allow the wound to recover.

MLM: Kamaaa inakidonda ipik olnjani? Do you put medicine to the wound?

RESP: Eee eya taata nekipuo ayau ilkeek lesipitali nekipik. Sometime we collect some hospital medicine and put in the wound.

MLM: Kengatitin aja oshi ipik olnjani? How many times do you put medicine in it?

RESP: Akainyang'u ake neeku enalo adol ilo tamweiai napik. I just buy it and every time I visit the patient I put in the wound.

MLM: Kekatitin aja oshi ilo adol ilo tamweiai? How many times do you visit the patient?

RESP: Eya nalo adol kila entedekeny ake eneeku egira aishiu neya nalo kila baada oo ingolong'i are, ong'wan. I might see the patient every morning. When he recover i might visit each every after 2 to 4 days.

MLM: Iata olnjani liudoki oltung'ani pee mening emion? Do you have anasthetic?

RESP: Maata. No I don't .

MLM: Kai ingo enigil oltung'ani oidikoki apa neriwoi engaina? How do you broke a hand of a person to reset?

RESP: Akawosh ake inewei teng'udi ometigilai ore ake pee egila naitoki aidikidik. I just hit the area with a stick and break it, then I reset it.

MLM: Ketii iltung'anak oibung ele tung'ani igilita? Are there people who will be supporting the patient while you are broking?

RESP: Eee ipaye amu eme toi naleng. Yes because it is painfull

MLM: Iramat oshi iltung'anak ootigilate arashu otuata ilmorlooshi? Do you treat cartilages?

RESP: Eee kake eeta sii eramatata enye enaa taata engung'u naa kayen tenjoni naudi tiabori naud teshumata pee etum oltungani atushuko engung'u. Yes but it has it own way of tying. The hide is toned at the top and below so as to allow the patient to bend the knee.

MLM: Kamaa enashoni nipik kenengine enake enengiteng? Is it a cow or goat hide.

RESP: Enengiteng ake. A cow hide.

MLM: Emintumiayaya oshi enenger? Do you use a sheep hide?

RESP: Ore apa enenger naa aikuntumia apa enaa olkodoro ina alo natii ilpapit, neeku meekure oshi kitum amu akemir iltung'anak indapan neeku mekitum enaake. The sheep hide was used as a matres the side with alot of fars. I don't get it now days because people sell the hides so i have to use a piece of matres.

MLM: Kamaa ilo kodoro kaireren openy enake aijoki iltung'anak meyau? Do prepare the piece of matres your self or you order the relatives of the patient to bring?

RESP: Nanu ake olilitu amu meyiolo ninje entoki napiki nanu ake oing'oru. They don't know the equipments to be used so I prepaired it my self.

MLM: Kaa rerenata duo oshi iasi yasa? Which prepairation do you do before treatment?

RESP: Akayel ake ilo godoro teilata eton itu aitumia. I just smear the piece of matres with oil before using.

MLM: Iramat oshi ingera naini eriuo ingejek? Do you set bones of infants babies with bowed legs?

RESP: Maramat oshi nena. No i don't treat them.

MLM: Kamaa taata engidonda nagira awotiwota kaingo? What do you do to an inflamation on the broken part

RESP: Ilkeek lesipitali ake apik. I just buy medicine in the hospital and put it.

MLM: Kai ingo enipik lelo keek, kaibukoki ake enake iata alpamba. How do you put the medicine on the wound? Do you pour on it or you have a cotton wool?

RESP: Akabukoki ake kake doorie pee atum oltung'ani oiwotiwote engidonda. I just pour on it and after all it is very rare to get such cases.

MLM: Keeta kulie keek lesipitali arashu kulie tokiting esipitali nintumiayaya? Is there any other medicine or equipments from the hospital that you used?

RESP: Kiata siyiok kulie keek loolmaasai lekintumia enaa ele shani oji elsayieti laa kakioju ake nendong nekironyoki engare inakidonda niitoi pii nemisho eng'wei. Naa intana ilo shani oshi kindumia, neya ake sii nekipupu nena benek nekipik engare ina kidonda. We also have some herbs to use like the "elesayieti" you pill it and grind it then you pinch it to get the juice the we put on the wound. It dries up the wound and prevent any wroting. We use mostly the roots of the herb and sometime the leaves.

MLM: Kamaa ele shani keer ilkur tengidonda? Does the herb destroy larvas when ever it develops on the wounds?

RESP: Eee ear naleng ilkur. Ore taata oltung'ani otoniko naleng itu ayakini netijing'a ilkur naa iloshani ake apik neer lelo kur. Yes it kills. For a patient that delays from coming to the treatment and he had the larvas on the wounds then when I put that herb it kills totally.

MLM: Intumia oshi engilang'et? Do you use x-ray machine?

RESP: Maata nanu engilang'et, akairapirap ake . I don't have an x-ray machine I just sence by using hands.

MLM; Kamaa napai eninepu ena nitu edany oloito idany abol, ata enidol ajo eturrumuo oloito enjoni? Can you open the skin on the broken area?

RESP: Aaaa mabol enjoni pii, akashor ake oloito oometushukoi ejing esidai. I do not cut it I just massage until the bone get into its position.

MLM: Kamaa ira toi oloiboni enake oltung'ani oomonisho tengoitoi ekanisa? Are you a religious healer?

RESP: Mara nanu oloongidong'i kake tenabak sii oltung'ani naomon Enkai meishiunye. Kake mara olekanisa, Enkai ake nanu asai. I am not a religious healer but I believe and pray to God.

MLM: Kanyoo oshi indai oltung'ani eniramat? How much do you charge a patient?

RESP: Aidai oltung'ani kitok engashe arashu ilakin are, ore tengerai naya embalelo arashu ingeek onom. Kake maya nanu onyoo ometaba nishiuu oltung'ani. For an adult I charge a cow or 200,000Tshs and for a child I charge a goat or 50,000Tshs.

MLM: Kekirusha oshi iltunganak? Do people escape from paying after they recovered?

RESP: Eee airusha kake mepuo oshi sininje adupo eitoki ake aagila neshukunye enatii amu manang'aa oshi inashoni hovyohovyo akael teilata napik enirobi. Kake teneshukunye inapa ake nanu aitalak majo aitalak ingumok amu ekwata apa. They they run a way but they do not win, they will come back again

with the same problems because I do not through the hide I keep it in a cold place. But when he/she come back I just charge the same I don't have to charge more because he ran away.

MLM: Kamaa toi imbelekeny toi oshi enashoni arashu ena kodoro? Do you oftenly change the hide or the piece of matres?

RESP: Meibelekenyi akaitusuj ake aitirish enesar engeju oo metaba neishiu oltung'ani kake ebik oleng itu itauni. I don't change i just keep adjusting or tightening as the patient recover but it stay for long time without removing.

MLM: Kanyoo duo indim atejo teramatata esipitali? What do you say about bone setting in hospital?

RESP: Meeta tukul enajo amu itu aikata aduaki kake ore oshi enaa enajokini iltung'anak njere ore iltunganak oyawaki sipitali ore pee ishiu neriwei ingejek kake metii aikata oltung'ani laidikidiko neeriwei engeju arashu engaina. I can't recoment anything as i have never witness any treatment from the hospital but I saw many patients treated in the hospital and they have lame legs or hands but to me it has never happen that I treated a patient and the legs or hands bends.

MLM: Kengejek toi ake oo ingai iramat? Do you only treat legs and hands?

RESP: Aaa aramat ake iltung'anak oogila ilaras oo engoriong kake maen amu meenai kake akashor ake aitorioo. Yes i treat also people with broken ribs and backbones although i don't tie but I massage to correct them.

MLM: Kamaa ele tung'ani oya engoriong, kamaa enishor keeta olkiragei oirag? How does the person with back problems stay while massaging.

RESP: Akajoki meiraga tengoshoke, naa kajoki meitoki aang'uyana, neeku akashor ake aigiligil omishiu. I tell him to sleep on his/her stomach and then I massage.

MLM: Kamaa oltung'ani oota shida engoriong kaishor kutwa? For a patient with back problems do you massage daily?

RESP: Aaa tukul akashor ake taata nebik ingolong'i are ore pee eeku ishiu neeku aitalam eya nebik ewiki. I just massage after every two days and when he is about to receover I increase the interval to a week.

MLM: Kamaa keeta eweji nemiindim aataramata? Is there anything that you can not treat?

RESP: Pookin taata eweji namaidim ataramata, elaa mayelo itu aikata alaikino oltung'ani, kake itu aikata abak elukunya arashu atua engoshoke. I have never treated head injuries and the inside the stomach.

MLM: Keyaa nijoki oltung'ani meshomo sipitali arashu likai dakitari looloik amu mindimu? I have ever refered a person to the hospital or to another bone specialist?

RESP: Itu aikata alaikino oltung'ani nashuk arashu ajoki shomo amu maidimu. I have never fail to treat a patient or refer to any where.

MLM: Kitu aikata kiakini oltung'ani otadanye elukunya? Have ever received a case of a person with head injuries?

RESP: Itu aikata ayakini oltung'ani otadanye elukunya, kake etii oltung'ani otanang'ayie olpikipiki negil eloito oshi lolkibelekenye orumita alairakuji, nalo ashor ore pee aidip najoki ewaita sipitali amu mayelo

enake etara oloito alairakuji kake itu ninje itoki aya nishiwuo. No although there was a person who had a bodaboda accident. I massage the person on the hip bone and after that I asked them to take him to the hospital to check if the bone has injured the kidney. They did not take and he has recovered

MLM: Kamaa ing'war duo taata pee kindimieki pee iasisho enaa ildakitarini lesipitali? Do you agree if your trained to work like hospital doctors?

RESP: Eee tukul. Yes for sure.

MLM: Keyaa duo nidol ajo etii nai intokiting naare pee kiretokini pee ilep eng'eno ino naleng? Do you think that there some ideas or equipments that if your provided with uplift your way of working?

RESP: Eee tukul amu epaasha sii engirapirapata oo engilang'et meetaa enaidimieki mailang'a neeku sidai oleng. Yes for sure because I just predict by using my fingers if I will be trained and provided with x-rays then it will be good.

MLM: Kakwa kibelekenyat duo idol ajo enibelekenyi nedupoi enasiai ino? Which changes do you think that when they carried your work will improve?

RESP: Maiba napai enatum endoki naitoki alang enashoni ai, amu ingaik ainei naatoi ake ingariaa amu maata naatoi inareta naadupo naleng. I just want to be provided with equipments to replace the local one have like the hide.

MLM: Keeta engai toki niyeu niliki iyiok? Do you have any more question or anything elso to say?

RESP: Eee amu ayeu naleng natum enaiko pee aitutumokini ilang'eni lesipitali pee atum eretoto teninje nedupoi esiai ai naleng. I just want that you connect me with specialist in the hospital so that we work together and my work will improve.

MLM: Keeta entoki nitu kingilikwani nenare duo nekingilikwanutwa? Is thre anything important we skipped from asking?

RESP: Elaa ijo meeta amu ijo ore nitolimutuo natoolua pooki ake enajokito ayeu naaretokini naleng. There is no more just requesting that I need support in my work.

MLM: Iata engerai ino ninteng'enita enasiai? Are you training your children about this treatment?

RESP: Mayelo amu itu aikata autaki kake autaki ake eneyeu. No because I have nevertarined but I will do it whenever they need it.

MLM: Kanyoo engipato ino talapa? How much is your monthly income?

RESP: Inoolong ake naramatisho natum nena. I only receive pay the day I treated some one.

Demographic information removed

TBS 3 - Arusha

Interview conducted in English and Maa, translated/transcribed by ML. Blue text added by EBC listening to the vocal English translations in the original recording

MLM: We are interviewing you because the knowledge you have on bone setting is important in helping people with broken bones.

RESP: True

MLM: Do you have any question before we proceed?

RESP: I don't have because you have called me so that you listen from me but i really appreciate this.

MLM: What are your first thoughts when first approached by researchers?

RESP: I really like because i have been approached by community leaders trying to document all that i do. I gave them information.

MLM: Which services do you over to your patients?

RESP: I always use hides in setting bones. I massage the broken arm and after that, i tied it with a piece of mattress then the hide. The piece of mattress prevent the skin from contacting the hide which might cause bruises.

MLM: Is it hard hide?

RESP: Is just a normal dry skin of a cow, i just make several holes on it and change after some time or if the arm swollen i released the skin.

MLM: Do you treat other diseases or you are just a bone setter.

RESP:I do the other thing that I do is helping animals during calving.

MLM: Also i set bones in animals.

RESP: I just massage using hot iron.

MLM: Do you treat long injuries that make people lame?

RESP: Yes i do. There many people taken to hospitals and after they recover they become lame. There relatives brought them to me and I broke them and reset them a fresh.

MLM: Do you always treat people with joints pains?

RESP: When they brought to me people of this kind I massage them correct the pain. Do you remember a young boy from your family who belongs to alaishoni? MLM: Yes. RESP: The boy was taken to various hospitals and no success and when they brought to me I treated and he recovered fully.

MLM: Do you treat leg or joint pains?

RESP: Mostly I treat those with broken bones.

MLM: When did you started bone setting.

RESP: Long time ago when I am a moran about thirty years ago.

MLM: Who teach you about this treatment?

RESP: Just God because there was an old man whom I was helping while setting bones and that where I learned to do this work at the time he become too old.

MLM: Were you related to the old man?

RESP: We were not related we are just neighbours living in the same big boma with multiple unrelated men on different sides. He did not teach me I just imitated from him.

MLM: Were you many while observing the process from that old man?

RESP: Yes we were many but I was the only one who took into consideration. When the old man died people started bringing the patients to me.

MLM: How many days did you observed the old man before you started doing it yourself?

RESP: I only observe three cases.

MLM: Did the old man trained you?

[TBS3.1 22:43] RESP: He has never trained me I just imitated from him. I just started doing it after I observed. The old man has two men, or his - two sons actually, and they never, they never do it. So he was the only person who really observe, wanted to do it and also the old man knew someone who wanted to know, .when he was older he was about to die he told the community that this, man, this little boy, is clever he can do it, so actually at the end he tried to refuse, he tried to say no, I don't know! And everybody say no, the old man said you know, you know, like an expert. And from there he started doing it.

MLM: How old are you when you stated bone setting?

RESP: I was about thirty years.

MLM: Where did you started bone setting?

RESP: I just started here with a patient brought to that old man who by the time was too old to perform the treatment. He told me to join the bone under his observation and when had completed he commented that I did it perfectly. From there I started setting bones.

MLM: Do you always depend on this work or you have other activities for your income?

RESP: No I have cows. It is also paying because an elder person pays a cow and a child pays a goat or sheep. But I don't fully depend on it because the patient are not readily available they come occasionally.

MLM:How many patient do you treat per year?

RESP: I don't really remember, there was a child who just released few days ago, there was also a moran and one woman. It is almost ten people in year.

MLM: Do go to far place to treat people?

RESP: I just work in this area. I went to manyara and from there I came across to a lady with bone problems and I treated her. There was one person who was an airtel tower engineer and he had a lame

hand. Some people told him about me and ordered that I should meet him. I discouraged him that broking the hand is dangerous perhaps. He said he is ready for anything. I broke the hand and reset again. He treats people in their homes.

MLM: How do you set bones?

RESP: When the patient is brought to me, I diagnose and set the bone. I give the person iloodwa, olmisigiyioi, oil from a sheep but I he will not be given milk any more.

MLM: How long do you massage the patient.

TBS3.1 [42:48] RESP: It only take like half an hour.

MLM: Do wrap the hide around the leg or hand or you cut two pieces and tie to the legs and arm?

TBS3.1 [44:50] RESP: One side of the hide has holes made on it and the other side is split so that each split get into a hole and when the leg is swollen i adjusted the hide.

MLM: How long does the patient remain immobile?

RESP: For a severely inured person he can stay for about a month. If not severely injured he can only remain for two weeks. For the patient with hands problem he /she can still walk.

MLM: Are you the one to prove whether the patient has recovered?

RESP: I just test him and when he showed that he has recovered I released him.

MLM: How many days do test the patient to prove his recovery?

RESP: I may stay with him for three to two days. when he is still sick I make sure that I am near to make sure that i rectify any abnormal occurrence. When the person recover I untie the broken part.

TBS3.1 [52:13] MLM: What does the sheep oil help?

RESP: The sheep oil is the first considered maasai medicine and it the first to be administered to the patient. The oil strengthens the bone and helps it to heal. He drinks it. Sheep oil is fat extracted from the meat.

MLM: What does the herbs help the patient?

RESP: These two herbs dry up the wound and prevent swelling of the broken part. But also iloodwa is herb for gonorrhea

TBS3.1 [56:00] MLM: How do you prepare the herb?

RESP: I just boil the olmisigiyioi (piece of a dry stem) to make juice from it and the patient drink it like water. For iloodwa (small fruits, found in mountains mainly) it is grinded, boiled, sieved, and made into a porridge from the juice and is given the patient. Olmisigiyioi is a very common tree in the area. Also feed them dry meat.

MLM: Why do you forbid the patient from taking milk?

TBS3.1 [59:30] RESP: It swells the broken part likewise soups. (Milk and soups are not good because they have too much water and they cause liquid to come out of the wound, which is not good)

MLM: How do you treat a patient with a protruding bone above the skin?

RESP: I just make a hole on the hide and the piece of mattress equal to that made by the bone so that to allow the wound to recover, to reduce pain by friction over the wound, and to make space for discharge from the wound to come out.

MLM: Do you put medicine to the wound?

RESP: Sometime we collect some hospital medicine (iodine or alcohol) and put in the wound.

MLM: How many times do you put medicine in it?

RESP: I just buy it and every time I visit the patient I put in the wound.

MLM: How many times do you visit the patient?

RESP: In the beginning, he visits the patient everyday to apply the medicine. When he recover i might visit each every after 2 to 4 days.

MLM: Do you have anesthetic?

RESP: No I don't . Everybody has to be strong.

MLM: How do you broke a hand of a person to reset?

RESP: I just hit the area with a stick and break it, then I reset it.

MLM: Are there people who will be supporting the patient while you are broking?

RESP: Yes because it is painful

MLM: Do you treat cartilages?

RESP: Massages tendon injuries. For injury in between the bones of a joint, it has it own way of tying. The hide is toned at the top and below so as to allow the patient to bend the knee.

MLM: Is it a cow or goat hide.

RESP: A cow hide.

MLM: Do you use sheep hide?

RESP: The sheep hide was used as a mattress the side with a lot of furs. I don't get it now days because people sell the hides so i have to use a piece of mattress. The mattress is old mattress and pieces of mattress he finds that people throw around outside (discarded).

MLM: Do prepare the piece of matres yourself or you order the relatives of the patient to bring?

[TBS3.1 1:12:57] RESP: They don't know the equipments to be used so I prepare it myself.

MLM: Which preparation do you do before treatment?

RESP: I just smear the piece of matress with oil before using. Apply oil to the side that is touching the leg.

MLM: Do you set bones of infants babies with bowed legs?

RESP: No i don't treat them.

MLM: What do you do to an inflammation/infection on the broken part

RESP: I just buy medicine in the hospital and put it. I have never before encountered a wound that gets worse and worse despite applying the hospital medicine (spirits and/or iodine) on it

MLM: How do you put the medicine on the wound? Do you pour on it or you have a cotton wool?

RESP: I just pour on it and after all it is very rare to get such cases.

MLM: Is there any other medicine or equipments from the hospital that you used?

RESP: We also have some herbs to use like the "elesayieti" you pull it and grind it then you pinch it to get the juice the we put on the wound. It dries up the wound and prevent any rotting. We use mostly the roots of the herb and sometime the leaves. We press the leave to get the liquid from it.

MLM: Does the herb destroy larvas whenever it develops on the wounds?

RESP: For a patient that delays from coming to the treatment and he had the larvas on the wounds then when I put that herb it kills totally.

MLM: Do you use x-ray machine?

RESP: I don't have an x-ray machine I just sense by using hands.

[TBS3.1 1:23:00] MLM; Can you open the skin on the broken area?

RESP: I do not cut it I just massage until the bone get into its position.

MLM: Are you a religious healer?

RESP: I am not a religious healer but I believe and pray to God.

MLM: How much do you charge a patient?

[TBS3.1 1:26:19] RESP: All payment after recovery. For an adult I charge a cow or 200,000Tshs and for a child I charge a goat or 50,000Tshs.

MLM: Do people escape from paying after they recovered?

RESP: They run away but they do not win, they will come back again with the same problems because I do not through the hide I keep it in a cold place. But when he/she come back I just charge the same I don't have to charge more because he ran away. Most patients run away, very few people pay. Elders bless the patient. The blessing is kind of security. If you are blessed, you are healed, and you don't pay the TBS money, then the patients will have their legs broken in the same place or another place until they come back and pay.

MLM: Do you often change the hide or the piece of mattress?

[TBS3.1 1:30:37] RESP: I don’t change i just keep adjusting or tightening as the patient recover but it stay for long time without removing.

[TBS3.1 1:31:19] MLM: What do you say about bone setting in hospital?

[TBS3.1 1:33:20] RESP: I can’t recommend anything as i have never witness any treatment from the hospital but I saw many patients treated in the hospital and they have lame legs or hands but to me it has never happen that I treated a patient and the legs or hands bends. He has corrected many ppl who were initially hospital patients. Therefore the people do not have trust in the hospital treatment, they will recommend my treatment. He cannot totally trust the treatment in the hospital.

MLM: Do you only treat legs and hands?

RESP: Yes i treat also people with broken ribs and backbones although i don’t tie but I massage to correct them.

MLM: How does the person with back problems stay while massaging.

RESP: I tell him to sleep on his/her stomach and then I massage. Restrict patient from movement

MLM: For a patient with back problems do you massage daily?

RESP: I just massage after every two days and when he is about to recover I increase the interval to a week.

MLM: Is there anything that you can not treat?

RESP: I have never treated head injuries and the inside the stomach.

MLM: Have you ever referred a person to the hospital or to another bone specialist?

RESP: I have never fail to treat a patient or refer to anywhere.

MLM: Have ever received a case of a person with head injuries?

RESP: No although there was a person who had a bodaboda accident. I massage the person on the hip bone and after that I asked them to take him to the hospital to check if the bone has injured the kidney. They did not take and he has recovered

MLM: Do you agree if you're trained to work like hospital doctors?

RESP: Yes for sure.

MLM: Do you think that there some ideas or equipments that if you were provided with uplift your way of working?

RESP: Yes for sure because I just predict by using my fingers if I will be trained and provided with x-rays then it will be good.

MLM: Which changes do you think that when they carried your work will improve?

RESP: I just want to be provided with equipments to replace the local one have like the hide.

MLM: Do you have any more question or anything else to say?

RESP: I just want that you connect me with specialist in the hospital so that we work together and my work will improve.

MLM: Is there anything important we skipped from asking?

RESP: There is no more just requesting that I need support in my work.

MLM: Are you training your children about this treatment?

RESP: No because I have never trained but I will do it whenever they need it.

MLM: How much is your monthly income?

RESP: I only receive pay the day I treated someone.

Demographic information removed

TBS 4: Arusha

Interview conducted in English and Maa. Translated/transcribed by ML

MLM: Ejoito toi aikioto duo pee kingilikwanishore iyie amu kidol duo aajo ore eng'en niata oo enaimaki iltung'an, naa eng'eno nagut oleng naretokito iltung'anak oogila neeku ninye pee kietuo akilikwanishore iyie. We are interviewing you because we understand that the knowldge you have is important for the born setting in kilimanjaro region.

RESP: Esipa. True

MLM: Ejoito keeta endoki nijo eton itu kingilikwanishore iyie? Do you have anything to tell us before we ask you anything?

RESP: Maata tukul. No I don't have

MLM: Kanyoo duo endoki niinter abiribir inidol ilaikeernok kiponu aikilikwanishore? What are your first thoughts when approached by the researchers?

RESP: Enaa duo taata nena nekingilikwanana? About the things you are asking me?

MLM: Eeee. Yes

RESP: Akajoki ake nanu karibuni amu ore nena pookin niyeu pee kingilikwanana naa kaaliki. I just want to tell you that you are welcome and anything that you want from me I will be ready to answer.

MLM: Kamaa ira oldakitari loo ilmasai kakwa ramatat oshi injo iltung'anak? As a born setter what services do you provide?

RESP: Ore naa peeku ina ore nabo naituruku, mara ninye nanu olmusomi metaa mooltung'ani laatuutaka kuna naasita, engai ake. Ore eenkai ore iltung'anak pookin oba ilataramata nishiutuo pookin

metii alatunuta. Ore ilooshomo mount Meru neshukuni nalotu nanu openy aidikidik nepuo nemetii olng'ojine, oo olotigile engaina, oo olotigile engeju metii, najo Enkai ake nairukoko enaa paa atuutaka tiatua kulo kumok amu mara sii olmusomi metaa maakatuutaki. Ore sii entoki oshi naitobirie enegila oltung'ani engeju naa kaapuei ake aaki injomboii tendaalo amu maata engaraha nairinie, nemaata empawoi akalo ake ayau tidialo. Nemaata si nena shomboi tiang amu maidim aitoripo iltung'anak, akaanyu ake ore pee ayakini oltung'ani nadamu entoki naitobirie naapuei ayaki teidie aa olishi gam lengarasha oyiata aa inoshi arash naibor esipitali, ee embaoi metaa ene ene netua tengaina (itodoluta entakule engaina) nayet, naeyel ashor,naibung kulo oik inji, naitorioo, nayet naibung'aki oltungani taata tioriong inji, nayet amatadua openy ajo etijing'a oloito enaa enapa nayenaa akaija (aitorioo) naa akaitabaki engaina enjata ene, naiatabaiki ene ore pee ashuku engaina aeniki engaina emurt omishiuu aleenoo amu akeye nebiki iwikii are nalak aing'uraa, neya nebeki iwikii uni nalak aing'uraa ore ake pee eishu ilapaitin aare nalak najo ake talaa inji ake etiu enaa apa. Ore engeju enene netua (etodolua olorroki lengeju) nayiataa, ayet amatadua ajo aitutumo iloik napik embaoi nabanji metabau ene, nebau ene(eitodulua engitanyanyiket teng'udi enye aiteru tengalo oldeima oo metabaiki alasaingat tengoshoke engeju) oo enapik metabau ene nebau ene (itodolua engitanyanyiket teng'udi enye aiteru tiabori engung'u oo metabaiki eniterunyie endap engeju kake tengoriong engeju) nayenaa naa taata enapaoi tene nayen tene (itodolua iwoitin neyenie enakeju natigile tengitanyanyikiet engeju enye). Nitoriori ena keju oo metaba nishiu ore kenya pee alak netiu inakeju enaa initu egila, oloyewaki mount meru, oo ilitu eyai. Neeku maata oshi injoi tiang naasie nena kake tenayakini nadamu naguatu ingeek naidikie. if that is the case, the first thing is that i am not educated therefore i have not gone through school and no one as tought me this, only ability from god's giving. Secondly all patients that went through my treatment has fully recovered and even those who firstly taken to mount Meru hospital fail in treatment and they were brought back to me and they all recover after my treatment and I can say it is only God giving that enable me to perfome all this because I am not a learned person therefore i did not learned. The things that I usually used to set a bone of a broken person are brought to me, i dont have them at home, they will ahve to and bring them for me, I dont have a peice of cloth to wrap on the broken part and i dont have a piece of wood they just go and bring it to me. I dont have a well equipment for this treatment at home as I can not keep predicting people to be broken, i just wait for the patient to be brought to me and that is when i order the equipments after diagnosing the problem, they bring me a bandage, white peice of cloth and a peice of wood. If the broken part in the arm is here.. (showing the arm), I just pull it, stretching it and while stretching the other person is pooling the person at his/her back, I keep on strecthing untill I see that the bone has return to its initial space and then i tied it and tie the arm on the neck with a rope and keep visiting to observe the progress. I sometime spend two weeks or three weeks and check to see if the patient has recovered. After two months I untie the patient and allow him/her to go. So generally I dont have materials at home to practice this treatment.

MLM: Kamaa napai enipal ena ramatata nindikidik iloik kakwa ingulie ramatat ninjo iltung'anak? Apart from the bone setting service, which other service do you provide to the people?

RESP: Ore sii engai eare, enejo engiteng eisho nelaikino aitau ilo ashe, naisuroki engaina atua engoshoke engiteng, nalo aipil ilo ashee amabainu naitadoiki engeene olgos lalashe nayetu omaitau nele engiteng amej tidie weji. The second service that I provide is helping cows during birth failure. When the calve inside the cow is having some mislocation that make birthing difficult, then I can enter the hand into the animal and correct the mislocation and proceeding in removing the calve alife by pooling it using a rope that insert on the neck.

MLM: Nemegor alashe engeene? Do the rope survocates the calve?

RESP: Megor amu aibung'ita. No it does't survocate because I will be controlling the rope.

RESP: Tenainepu engiteng natunuke enguset napik engalem atua engoshoke nalo apolos inewei negol nadany naitau ake alashe nelo ake engiteng amej. Tenainepu iltung'anak oruoyo ingonyek, naa oltidu ake aing'oru naoshoki inewei nerok teina oong'u napolos naibukoo inakipai piu namunoki emakat sapuk napuyiapui nenya tukul nanyinyiaki nenya tukul neyieng'i enger neishori eilata oree pee eripunye inewei natopolosa neishiu nerisoi ingonyek enaa nanu ake taata kake mitoki enda adolisho kake mitoki aasujare amu akenya inamakat omeishu inatoroni. Naidipa aatadanya iltunganak obaya oong'wan naa kerisio ake taata ingonyek pokira, netii nabo kinyi natodolishe taata. Neeku ore taa nena ramatat nee Enkai ake natuutaka amu mara sii olmusomi lenana tokiting. Neipurupuro sii engiragata alashe enaa enoshi napuku engirragata alashe, nelaki olnjoni, neilang'ilang'i engare nairowua peno, neyauni esabuni, nelaki taata olchoni lengiteng, naibung'aki iltung'anak enatoki oobaya tikitam arashu tomon naisuj nainyaaki atua engiteng nelo ake inakiteng aitoki aisho.If i found a cow with a bis ceas in the womb that make birthing difficult I can operate it and remove the calve savely. If a find a person with the pupil inflamation or swelling then I torn the pupil using a nidle and remove the eye content, then I put a litle amount of soda ash, a sheep is slaughtered and the patient is given sheep oil and when the person recover the shape of the eye remains although a big number of them loose their vision but bose eyes will be having the same shape despite one loosing its seeing ability. I have already treated four people and there is one hwo has gain vision in the defected eye I think because i treated here at a younger age. Only God has shown me this as i have never gone to school. Also when the uterus of cow came out I was it with worm water and soap and return back into the animal and the animal countinue being fertile.

MLM: Itolimuo nena oshi pookin nias enake etii enitudung'oyie? Did you tell us all the services you provide or there is more?

RESP: Ajo ore enabayie naa ninye alimu naa ninye alimu, alimu ajo ore taatoi lelo tung'anak lataramata aimaata ake kumbukumbu kake ataramata ake ninye iltung'anak oogiroo mia mbili, itu aikata igili aaya iltung'anak tekulo oshon ainyaya idialo nanu ake eyakitai tene. The only thing to add is that I have just treated more than two hundered people although I have no clear written records. People are no longer taking people to hospital all this neibouring, they just bring them to me.

MLM: Kena toi ake esiai ino nitumie endaa oo ingera? Is this work the only source of income?

RESP: Maata engai ena siayia ake. I have no other work only this one.

MLM: Miramatisho? Do you keep animals?

RESP: Aramat ingishu tukul. Yes i am a pastoralist

MLM: Kebaa engata nitaramatishe aramat iltung'anak enaa aldakitari? How long have you been treating people as bone setter?

RESP: Elaa elakwa oleng. It is a long long time.

MLM: Ira olmurrani? Is it when you are a moran?

RESP: Mara olmurani ara ake olpayian, aiterua oshi aaramatisho 1987 inakata eutoi ilkorianga kunyinyik. I was not a moran I was just a junior elder in 1987 when the younger Ilkorianga are being born.

MLM: Kai apa inguna oo interu taramata iltung'anak oogila? How did you started treating broken people?

RESP: Ingishu apa aitera airuburub naagila, ore ake pee eshiu engiteng nairuburuba, nang'amu taa taata oltung'ani ledukuya otigile neji eyaki ilo payian amu eyiolo adikdiko amu idikidik oshi ingishu. Najo ake indikidiko ilo nishiu nitu eng'ojinu nalotie ina naidipa taata aidikidiko iltung'anak mia mbili. I started setting bones in broken animals, after that I started driving that idea to people after I so that the animals are recovering. People brought to me the first broken person assuming that since I set animals bones than i might be able to set people as well. After I tried that one he recovered and the trend continues and I have already set more than two hundered people.

MLM: Oo inang'eno nindikidik ingishu, kang'ai apa nekinteng'ena? Who showed you the skill of setting animals bones?

RESP: Enkai ake naiteng'ena amu meeta sii oltung'ani laiteng'ena akegila ake engiteng naidikidik enaa duo pee ara alaramatani. It is just God who showed me this, I just started settinng animals as I am a livestock keeper.

MLM: Ketia kata apa intera aidik ingishu naagila? When did you started setting broken animals?

RESP: Aiterua apa ara olmurani, aalo ake ilarin osom oima. I started when I was a moran, more than thirty years ago.

MLM: Itiaka napai iyiok ore eng'eno niata naa Enkai ake nekituutaka, iindim ainasaki iyiok enikunakino ina utaaroto nekituutaka Enkai? You told us its God who showed you all this, can you tell us how God showed you this?

RESP: Mayelo amu Enkai ake naishoo engolon engoshoke naa ninye naidimie matetema intikitin. I don't know how but it is God who gave me courageous in trying things.

MLM: Ketia apa intera aramat oltung'ani linterua? Where did you treated your first patient?

RESP: Tena murua ke. In this area.

MLM: Kamaa eningo oshi enindikidik ingishu neija ake ingo enindikidik iltunganak? Do you set peoples bone the way you do to animals?

RESP: Eee neija ake aiko. Yes I do the same.

MLM: Ketii ingibelekenyat enaa eniramatita iltunganak? Are there changes as you practice bone setting?

RESP: Eee etii ingibelekenyat, ore apa tedukuya nemapik enjata naado kake ayeuo oshi taata adol ajo enapik enjata naado naiteru toldeima oo metai engalo endapa, tengohoke engeju naa kesieki oltung'ani ashiuu. Yes there are changes, in the begining I dont use a very long piece of wood, but as time goes I discovered that if use a long piece of wood the patient recovered quickly because it restrict movement.

MLM: Keyaa netii olikai tung'ani oota ena ng'eno tenalatia ino? Is there any other bone setter in this neighbourhood.

RESP: Metii. No

MLM: Inoto aikata engiteng'ena ena ng'eno? Have you ever received any training about this training?

RESP: Itu aikata atum. Aata taatoi alaretoni lai oshi laaretoki, netayiolo sii ninye enasiai. Amu ninye ake egol oshoke oleng. I have never received any training. I also have my personal assistant and he has started knowing how to perfome this treatment as he is the only person who is courageous.

MLM: Kalalashe lino toi? Kai ibaikinono? Is he your brother? How do you related to each other?

RESP: Eee alalashe lai, aikira ilaangainito. Yes he is my brother from another mother.

MLM: Kai inteng'en oshi ninye ena siai? Do you usually train him?

RESP: Maiteng'en akaibung'a ake aduaki oo metayiolo. Nidipa ake sininye aidikidiko iltung'anak oongwan nishiutuo. I dont teach him, he has just observed me doing it until he become an expert and he has already treated four people and they were successful.

MLM:Iindim ainasaki iyiok eningo enindikidik oltung'ani otigile? Can you please tell us how you set the bones of a broken person?

RESP: Akeyauni ake oltung'ani, nang'as ayeleu ajo kanyoo nataase, neyauni eilata nayet taata enaina natigile ashor naa tenetii taata oloito oyeu neisegela abaina, nairony taata airony, nashoroki esidai, naiteraki aitaman engarasha pee enaen too ingeek nemedot ingeek osesen, ore sii kuna keek nairin tengarasha pii pee mear osesen, naa inakata esieki oltung'ani ashiu. Naa ore pee aidip aateena engaina naeniki emurt pee meitoki engaina aipusho oo metaba neishiu. After the patient is brouht to me, I diagnose to find the problem. When i discover a dislocation or broken bone, then I message it using oil until the bones came to its position. Then after the bones has come to its position I tied the arm or leg with piece of soft cloth so as to prevent any scraches from the wood that is used to tie the broken part. Also putting a soft cloth or wrapping the pieces of wood with a soft piece of cloth before tying will ensure quick recovery. After tying the arm with this pieces of wood, i tied the arm firmly to the neck to restrict movement and ensure that the arm recovered quickly.

MLM: Kai itumie engara niyenie engaina oo kuna keek? Where do get a piece of cloth and the piece of wood to tie the broken arm?

RESP:Olbiranketi oloshi loolmaasai ninye ake kipolos nekirinie ingeek nekienie engaina. Ore pee egila oltung'ani emolingash naa akayakini algam oloshi oirinieki intokitin. Naa kagwatu ake engiti shata dapash natashaki inewei emolingash natigile nairony akeduo emolingash ore ake pee etutum ena shata inewei natigile nayen oltung'ani aimie engarasha ironyi pokirare aitorio nishiu ilo tung'ani. I just make piece of cloth by torning a maasai shuka and use it to tie the arm. And if the person has a broken clavicle, I use a black big selotape and make piece wood equal to the size of a clavicle, then I press the clavicle bone using the piece of wood and then tie using the selotape. After that the person shoulders are tied using a piece of cloth and the person recovered.

MLM: Kebaa endapasho ina shata? How large is that piece of wood?

RESP: Ore endapasho in shata naa eba ake kulo kimojik aare enitash. Aata sii oltung'ani atara engarim, neye iltung'anak aare neleku iltung'anak aare, olotoosho engarim nelo apetaa osoit, neosh osoit abelu eloito nengalo oldoima. Naibung oltung'ani aitolok toongej ainei nairony orony lenye nairony ingejek enyana najo ing'urai oloito loloroo otebele nashor ashuku, ore ake pee ashuku nepaasha iloik neitoki ashum engutuk ilo oito. Ore ake pee adol ajo etaa neija naguatu enjata dapash, naen aimie enewei oshi neeim alalem, neirony aladuoo oito otushumunye naen toonjot pookin nibooki, ore taata ilo tung'ani eshiuo eshomo engang enye. The size of the wood is just the size of two fingers put together. I had a

case of a person involved in an accident. Two people died and one was thrown by a car hit on a stone and the hip bone was broken in its lower part. I lay the person on my thighs and press the shoulders and the legs until i discovered that there was a mislocation in the hip bone. I massage the patient and tied the broken bone with piece of wood and the person has recovered and went back to his boma.

MLM: Kanyoo pee irraje oltung'ani toongejek? Why do you lay the person on your thighs?

RESP: Akajo pee adol ewoi netigile esidai amu akairony orony oo ingejek. Inorder to see the broken place as i will have to press the legs and the shoulders

MLM: Kai toi itumie imbaon nienie iltung'anak? Where do you get the piece of woods?

RESP: Akayakini ake toowoitin neshetishoreki kuna dung'ot oo imbaoi nalotu aguat esidai. They just bring me some left overs pieces of woods in the building sites and I smoothen it myselve.

MLM: Intumia oshi ilkeek pee mening oltungani emion? Do you use anasthetic ?

RESP: Maata tukul ilkeek akeng'iri ake oltung'ani, kake ore ake pee aidip atabaa naisho ilkeek lengop. I dont have anasthetic at all, the person will have to tolerate but after treatment I gave them some herbs.

MLM: Kebaa engata nintumia enishor oltungani ewoi netigile? How long do you massage the patient?

RESP: Meya engata naado, ildaikani okuni oo metabaiki imiet it doesnt take long time, three to five minutes.

MLM: Kejiaa lelo keek linjo oltung'ani ildipa aataramata? What is the name of the herb you give the patient after treatment?

RESP: Eishori iloodwa neishori olmisigiyioi. The patient is given iloodwa and olmisigiyioi

MLM: Kai oshi ingo enindobir lelo keek? How do you prepare those herbs?

RESP: Akaidip ake aataramata oltung'ani, neyauni uloodwa neidong'i neitushuli oo olmisigiyioi neyeruni ore ake pee ewoku adoru, nesili, nepiki kule nepiki eengwa neyeruni euji neishori ilo tamweyiai. Ore pee emutu akateyiang'aka naa eleshani eok aitaa engare enyaita ingiri kake meitoki aok kule. When i finish treating a patient they bring iloodwa and grind them, mixing it with olmisigiyioi, then cooked until the mixture is red in colour, they make porage using maize flour and it is given to the patient. And if the sheep is slaughtered for the patient, then he/she uses the harb as drinking water and the patient is phobided from taking milk

MLM: Kaa rubata ekulo keek oshi iltumiaya? Which parts of this herb do you usually use?

RESP: Ore olmisigiyioi naa embinas ake edung'uni neyeri naa ore too iloodwa na lelo andarara ake lenye eyauni neidong'i neyeruni. For olmisigiyioi i use the stem and for iloodwa i use the fruits.

MLM: Imbelekeny toi kunatokitin niteenie oltung'ani enake aketoni ake omishiu? Do you always change the piece of cloth and the piece of wood that you used to tie the broken part or you just leave them until the patient recover?

RESP: Akaleeno ake pee enesarari enjan nelolong'u nena keek nalak naigilie ake aen. I just keep checking so that when the broken part reduce in swelling then I re-tie again

MLM: Kenena ake ingilie enake kulie? Do you re-tie with same same materials or new one?

RESP: Nena ake kake aisuj nena karash neitaari engolong penyo naigilie. The piece of clothes are washed dried and then i re-tie the patient broken part with the same.

MLM: Kebaa engata naya oltungani oo mishiu? How long does the patient take to recover?

RESP: Eya neishu oltung'ani alapa obo, obo oenusu kake meid ilapaitin aare. The patient might take up two months to recover

MLM: Keishori toi oltung'ani eloolo enakata emoi? Do you allow the patient to move while stilll sick?

RESP: Elo engang enye amu maata enapik, neeku nanu ake eyai tendukutuk mashomo aaleen. Nemeiruusini pee elo omishiu kake ore olengaina nepuku ake alo. The patient is taken back to his home after treatment, then i keep visiting him, they take me with a bodaboda. The patient with a broken leg is not allowed to walk until recover but for a patient with broken arm is allowed to walk.

MLM: Kekatitin aja oshi kiyai pee ilo aleen? How many times do you visit your patient?

RESP: Alo aleen terishata oo wiki are, ore kila baada oo wikii are nalo aleen. Naa ore enaaliki njere eshiuo ele tung'ani naa kadumu lita tatu engare najoki metudumu ayukuyuk ore pee mening'o emion nadumu lita tano engare neiko ake neija ore pee mening'o nadumu lita kumi najoki iyukuyuko ore pee mening'o emion tengaina najoki meshomo amu ishiuo nalak ina aina. Ore enaa engeju naa akejoki shomo atua kumi nelo neshukunye, najoki shomo siyie hatua ishirini neshukunyee, naitoki aajoki meshomo eneedo penyo ore pee eshukunye naikilikwan enake eton ening'ito ore pee meekure ening'ito nalak. Kake ore eneeku ajoki meshomo naa akang'as atau enashata naado. Every after two weeks. For arm broken patient and inorder to prove whether the patient have recovered, i just take three litters of water and allow the patient to lift and aske if he/she fill pain, then five, then ten if he/she do not fill any pain then I release him/her for he has rcovered. For a kleg broken patient, I test him by telling him to walk a certain distances if he can not complain pain then he recovered. Before allowing the patient to walk I remove the long wood that was used to tie.

MLM: Kamaa enindip aatetema ilo tamweyiai niyolou ajo ishiuo intoki ake aasujare aaku injo ilkeek? Do you give the patients any medication after he/she has recovered?

RESP: Maitoki asujare tukul. No i do not make any followup any more.

MLM: Kamaa enedany oloito enjoni, kai ingo eniramat ine netadanya oloito? How do you treat wounds that result from the bone broking the skin?

RESP: Ayelo moobo tung'ani atadanya oloito enjoni naramat. Akaisuroki openy olkimojino nairapirap aitau lelo oik oo tararate oomaishu napik oloshi shani oitokitok, najoki oltungani meteyiata inakeju nairony nanu ele oito oomitishukoi enaduo owoi enye. Ore ake pee inyaakino oloito ewei enye narip kake aitalam eripata pee atum eweji napikie olnjani. Ore pee aen ena keju nemayen inewei netadanye. Ore tenaa kinyi inewei netadanya oloito nadany tengalem aitalala pee atum aitau lelo oik oo tararate. I had some cases of this kind, i just enter the finger into the wound to remove small bones which might have been left into the wound. After that i massage the protruding bone until it gets in its position. After the bone has enter its position i sitch the wound but leaving gaps to ensure that there is a place to put the herbs through. Even if i will have to tie the place i must skip the wound. If the wound is small, then i will have to enlarge it my selve with a knife so that i can get out the small pieces of bones.

MLM: Iata toi inoshi tokitin naishopi too ingaik ebakishoi? Do you have gloves?

RESP: Maata tukul akaidipi ake naisuj ingaik. I dont have i just do bare handed and after that i washed hands.

MLM: Kanyoo oshi iripie inewei netadanya oloito? What do you use to sitch the wounded part.

RESP: Osindanu oshi lekiripie injashuri oo enoshi usi olbene lesukari arashu lolmushele oo enoshi naripieki ilkarash. I used a needle we use to sitch bush knifes pods and the string that is used to make clothes.

MLM: Irerenene toi ina usi enake aitata inoshi nainyang'uni tolduka? Do you have the string with you or you buy it in shops during treatment.

RESP: Enelotu oltung'ani meeta nepwei ayau. When the patient is brought and need to be sitched, then i send people to buy for me in the shop.

MLM: Kamaa iloshi keek lesipitali lipik, keyie toi opik openy? Do you apply the hospital medicine by your selve?

RESP: Akepwei ake ayeu ore nanu atibirita nanu oltung'ani neoshi endukutuk nepwei ayau. Napik nanu naitodol ilo tung'ani aata altamweiai pee emooki apik. The just go and bring them to me i apply them and show the relative of thepatient on how to apply the medicine.

MLM: Kanyoo toi ipikie? What do use to apply the medicine on the wounds?

RESP: Inasiote ake inabilbil natii anjani ninye apikie. I just pour over the wound using the bottle lid.

MLM: Kekatitin aja ijoki ilo tung'ani metamooki apik? How many times is the medicine administered or applied?

RESP: Eya nepik oong'wan ashu mbaka oometadua ajo eyeu nejing ingimek, neisuj ore pee etoi nepik ilo shani, neeku eya nebik ingolong'i ogw'an, ile arashu iwikii are. Four to six times but the interval also increases as the patient recovered

MLM: Kamaa toi enien kaing'waa elusie tineu neya oltungani? Do you leave the wound untied when you tie the broken bones?

RESP: Akaitapaash ake enjata inewei neya oltungani nayen tiai woi. Yes i dont tie the wounded part.

MLM: Kamaa iltung'an ooye imolooshi kai ingo? What do you do to people with broken cartilages?

RESP: Aata apa oltung'ani otabatate nedung'o ena morloo naibung'ita oloito lengung'u, netoi ina morloo pii nebik ilarin okuni. Nabaya ake najoki endeyieng olkine tene kake mesuokini, akerei intare linga ore ake pee eirowuaju, neshukuni najoki esarisara enteyieng nadanya engoshoki tiaraka atau engonyori naisuroki engeju oomeu shumata engung'u nemuk enakima enewei oo metanana ina morloo apa natoyuo enaa engata naba esaa. Najoki iltunganak meibung'a oltungani nanyor enewei neya apanya teng'udi, neishir oltung'ani ajo uui, naara ometalepori nelo oloito ajing enapa wei netii temusano, nepiayi engaraha naen enakeju teabori eloito lengung'u naimie shumata engu'ngu, neironyu inaarasha kitoni, nekiok imotori ina kine oo maidipa, ore pee ekenyu paalotu nanu engop ang, amu tenduimet ashomo abak ele tung'ani, ore ake pee eimi iwikii are nashuko nalo agil aing'uraa, najo shomo ingila shomo ing'urai etalepoori enapa weji nejing oloito ewei enye enaa apa ake temusano. Nejo ake ingila

iishu alapa nalak neroro engop nelo ake taata enaa iyie. There was a case of a patient with a broken knee cartilage and stayed for three years untreated. When i found the patient i ordered a goat to be slaughtered during day time aftergrazing for some time. The after slaughtering I quickly took the stomach of that goat while still hot and the patient entered his leg into the content of the goat stomach. The cartilage is soften by the temperature in the stomach content, then after that I massage the knee with smooth stick untill the cartilage stretches to its normal position then I tied it. That patient has fully recover and walked properly.

MLM: Kai toi inguna ajo eninjopoki ena onyori enakeju nelak ena morloo natumukute? How did you know that keeping a broken cartilage inside a stomach content for sometime make it tender?

RESP: Ake ore apake ninje iyiok ilmaasai tenikiar nai taata olkikaret nekigilakino naa kekiyiang'akini enger naa pookin ake duo entoki nayieng'ieki naa ele kikarer ake duo eji pee itaunyeki. Neyieng'i ake neitauuni engonyori neudi nipiki inakeju nekitaara olkikaret nemuka engata naado penyo, ore ake pee intai ade iropija ena onyori ore taisere nepuk aladuoo kikaret ata enake inji eba, nayelo naa ajo itanana imorlooshi. It is common among us that when you are injured by a thorn and remain in your flesh and difficult to remove it, we slaughter a goat or sheep and the injured part is put on the stomach content for sometime and then will protrude out after one day. We also know that doing this make the cartilages tender.

MLM: Kamaa oltung'ani oya irrubat enaa engoriong oo ing'ung kai ingo enerimat ilo tung'ani? How do you treat people with dislocated joints or back pain or knee joints problem?

RESP: Ore enajoki lautaki naa akajoki shomo teyiang'a enger aitau enger duo taata naata eilata ore nai taata paa ilkoboi oongwan itawuo ena ker, niindong iloodwa emborei oltumberi, ore ake pee ildong, ore ake pee idotu enamoti oo irongena, ore ake pee iet nibukoki kulo oodua atua ena ilata, neitokitok adoku adoku neeku aiyopiyopito ometushukoi ibibia kulo oodwa, neeku ena ilata iok enaa iyier endapa arashu euji arashu engai daa ore ake pee intumia ena ilata oo ilodua neishiu engoriong pii. I only give the person an advice that he should slaughter a sheep a fat one. Rost the fat about four litters then grind one kilogram of iloodwa then mix with the oil while still hot. The mixture is used by puting two to three spoons of it in foods or porrage. After using the oil for sometime the patient recover his/her back pain or knee joints pain.

MLM: Kai iko kulo odwa pee eishiwunye oltung'ani? How does iloodwa hill the person?

RESP: Akelo aitobir osarge losesen, neor osesen aoru ilo minjaloi lekigira aitesa. It cleans the blood and improve immunity.

MLM: Kanyoo oshi eretoki iloodwa arashu olmisigiyioi oltung'ani otigile? What does the iloodwa and olmisigiyioi help in a broken person?

RESP: Akepuo oshi aibibi olbaye tiatua osesen nemitoki inewei netigile oltung'ani aajei, neisho osesen engolon nemeitoki likai baye ayepu inagilata. Niitoi osesen. It dries up the wound and prevent swelleng. Also increase immunity and avoid other side infections.

MLM: Iramat tooshi olbaye otumusana enaa taata oltung'ani otigile neishuu kake etaa maima neng'ojunu ishiwuo? Do you treat long time injuries that make people lame although there is no pain?

RESP: Eee tukul, aramat, enayakini oltung'ani, amu eya neyai oltung'ani mount meru nemeyiet naatoi oshi ninje engeju ekemuraa ake duo ninje tesimiti naa keya apa netabatate oltung'ani nepaasha iloik,

neya nisho engutuk nabo oloito nejing atua entolit neimu olikai oito teshumata neeku naa meitoriori engeju ilo tung'ani. Ayakaki iltung'anak aare otiu neija nabo naata ilarin oopishiana oo enaata ilarin okuni. Naa ena naata ilarin okuni aiteraki ayaki, neishiwoko kake ng'ojine, neitabauni ake naing'oru osoit lijo engii nayau olninto natashaki engeju enakii, naosho tele ninto oo matigila nashor inakeju aayet nashor pii nabaraki aen neng'ejuku olbaye ore ake pee ishiu neeku akelo ake oltung'ani enaa iyie. Ore ake pee ishiu nejo kulie tung'anak etii sii ninye enaang natii ene naata ilapaitin kake akaalejaa, amu atiaka maitieu atabaa oltunga'ni apa oidipa apa aishiu, naajokini enaijo apa kitoning'o ajo itabaa oltung'ani oishiwuo engata naado netosidana? Najoki kelarin aja eeta ena inyi? Neji ilarin aare ninye kunyi ilarin alang enapa. Najo shomo ing'urai enaijo torrono. Najoki intewuu enagil? Neji eee. Nepwei ariku oltakitari oyeu aud osindano oshi lemion, ore ake pee eud nagil engeju nashor aitorioo naen aidikidik enaa enaiko oshi. Naa akepiki iltooi are lengare epiki miboi enakeju pee memerregela oo metaba neishiu. Ore kenya pee alak neishiuu nelo oltung'ani esidai. Ore ake pee aidip naaliki ajoki ilarin naapishana eeta kake akejo duo pee mapal. Yes i do treat. There are several cases of people taken to mount Meru and there had a POP on their broken legs and they recover but the leg is no longer straight. The problem with mount meru hospital is that they do not stretch and massage the patient leg. The patient might have an overlaping bone due to the accident. In this case the patient need to be massaged to make the bones location correct. Two patient of this case were brought to me one with three years and one with seven years I broke them. The one with three years had a lamed leg, i broke it using a hammer and reset again he recovered fully and walked like the other normal person. The other person was a lady who remain with a lamed arm for seven years after a POP was done. The relatives of the lady lie that the arm has just stay for two years, then I order her to be brought to me and ask them to bring the hospital doctor to administer anasthetic . after that, i broke the arm and reset again the lady recovered fully.

MLM: Kamaa ingera naini eriuo ingejek iltorioo? Do you corect bowed legs in infants?

RESP: Itu aikata aporoo. I have never tried.

MLM: Kama ewei nitu edany oloito onjeno, idany pee ejing olnjani? Do you always cut or make a hole on the broken part so that you put the bedicine on the broken part?

RESP: Madany tukul. No.

MLM: Kamaa oltung'ani enegila itudung'oyie aikata engaiana arashu engeju enaa duo ijo meekure ake esidanu? Have you ever cut any of your patients arm or leg thinking that he/she will never recovered?

RESP: Madung akata nanu. Ayawuaki apa aikata oltung'ani loo ilnjaga otejoki edung'ori engaina tesipitali nejo maadung'i, neing'oru oltung'ani lengop ang otii ilarusa neriku naaponunui aya nalo ainepu tepanadol narik engaji naji enemokoro naitorioo nashorn abak najoki tipika engaina erishata oo ilipirai aare ootii engare pee memeregela enirura nejo ake iishu ilapaitin aare neishuu pii. I will never do that. I had one case of a chagga patient where by the hospital decided that his arm must be cut. He reach me through a friend and i treated him and instructed him to keep his treated arm between two backets of water to avoid mobility. After three months the person recovered fully.

MLM: Ira toi olabaani laijo oloiboni anake olekanisa? Are you a spiritual healer?

RESP: Mara oloiboni nemara iloshi lekanisa tiatua ingishu ake atubulua. No I am not a spiritual healer.

MLM: Keltung'anak aja itaramata tiatua alari obo? How many paient did you treated for a year?

RESP: Kumok naleng, aiwore nenye tanakata iltungan oyena lemelaa, aare otii kiserian oore otii loongiito, ore ilatalaa telaapa nabaya ake ile. Neeku alo iltung'anak tomon talapa obo. Very many just in this days four people are under observation and a week ago six people are released. So just in amonth about ten patients.

MLM: Kanyoo oshi iltalak iltung'anak enibak? How much do you charged per patient?

RESP: Engiteng elak oltung'ani kitok naa embalelo engerai. One cow for an adult ane one goat for a children.

MLM: Keyaa duo oshi nekirusha iltung'anak? Do people sometime escape from paying after they recovered?

RESP: Aaa tukul eya akeduo netii ilemeeta neemoni meing'oru ore pee etum naisho. Ashu etii apa oltung'ani latabaa najoki miok inaishi amu mayeu kake aepurroo enaisho, neiruj ilkuru inewei neya nedumuni neyai tengeru neji elang'a nedoli ajo edikidikote iloik neisuji nepiki olnjani omeshiuu, neyauni olikai tung'ani oungwaa ina sipitali naajoki kitadua oltung'ani litabaa nishiwuo ninye pee kiotuo, nabak ilo neshiu. Ore alapa tung'ani neisiku tesipitali nesujuni neponunui aidai emilion, najo nanu tusuja nelo ajo meeyie lekindikidiko sipitali naishiunye. Ore oshi neosh endukutuk negil, neponu ilalashara lenyanak ajo kinjo iyie engiteng ino amu alapa mukosi oineputa ele tung'ani. Eton taatoi taata mereuno ina kiteng kake eishoruni naake. No at all just sometime delay in paying. There was one patient whom i treated and fobid him from taking alcohol but he ignored and did not recovered. He was taken to the hospital while the leg is still tied and the hospital just provided medicine as they said i did it correctly. The same person escaped from the hospital without paying bills and and the hospital follwed him he paid a bill of one million Tshs. After sometime he had an accident the same place is broken again. His relatives came to me requesting to pay the first bill because it was my right. I treatd again the man and he recovered.

MLM: Kamaa eramatare inyi oo enesipitali kaa napai iyie indim aatejo? Which treatment do you recoment is it the traditional or the hospital one?

RESP: Kaata oshi enatetemakine tesipitali metaa akayiolo? Did i ever tried any? I don't think so.

MLM: Matejo Kake kaa duo iyie indim atejo ninye esidai? But which one dou think is the best?

RESP: Mayelo katukul kake kumuk akeeny ilayakini nanu oing'waa sipitali naigil alotu aramat kake metii nanu alatalaikine ataramata neyai sipitali. I dont really know, but there are many patients that were brought back to me from the hospital but i have never refered any to the hospital.

MLM: Kakwaa tokitin esipitali oshi iltumia? Which hospital equipments do you usually use?

RESP: Embandage, oloishi shani loolbaa, oo enoishi arasha sapuk naenieki ingidondani. I use a bandage, iodine and plasters

MLM: Ketii entoki nemindim aataramata? Is there any case that you can not attent?

RESP: Maata nanu entoki nemaidim aataramata. I dont have a case that i can not attent.

MLM: Itiaka akata oltung'ani meshomo sipitali arashu enetii olikai tung'ani aria amu maidim ataramata? I have ever refered the patient to the hospital or to anaother specialist like you?

RESP: Itu aikata nanu ajoki kake eshukuni tesipitali. I have nevered refered although i recieve a number patient from hospital

MLM: Ketii kulie ang'eni looloik eletiren linyi ashu ena latia inyi? Are there any other specialist in this area or your neighbouring?

RESP: Nanu ake oo ele oshi laaretoki amu ninye egol oshoke. I am the only one and my helper here who is courageous.

MLM: Kamaa napai eneuuni oltung'ani letaa mindimu indim aatiaki meshomo sipitali? What if a patient is brought and you can't treat, will you refer him to the hospital?

RESP: Meteleku aa alaata engai moyian kake wore akeninye oletaa aketigile naa kaiteruoo ake, ore ninye olotigile engoriong naa enjata dapash ake aing'oru oo olbiranget naen ele tung'ani tolbandage sapuk aen angoriong neitoriori. Neeku meeta oltung'ani otigile lemaidim aidikidiko naa alapa obo ake etobiko. May be a patient with other health problems , but for those with bones problem i can do it, even a person with a broken backbone i can still treat.

MLM: Kamaa napai enitum enekinteng'enieki imbarakinot esipitali iiruk? Will you agree if you are trained on the hospital procedures?

RESP: Eee tukul airuk. Yes I agree.

MLM: Keyaa duo napai nidolita ing'orikinot nekimitiki ias intokitin kumok? Do you have challenges that are hindering your ability in working effeciently?

RESP: Eee injomboi ake maata enaa nena esipitali kake ore oshi enayaki oltamweiai naa akaing'or nayelou iltokiting naayeuni paaitumia. Yes i am lacking those hospital equipments.

MLM: Keeta endoki nitu kijo nanare duo nikitejo? Is there anything we did not ask which you think we could ask?

RESP: Metii tukul. No at all

MLM: Keeta enduki neton iyieu nijoki iyiok? Do you have any question?

RESP: Maata tukul. No

MLM: Keeta engerai nijo inteng'en ena siai? Will you train any of your child about this treatment?

RESP: Maata nanu oltung'ani laiteng'en ilo ake laa ninye odol openy. I will not teach any body except any one who need to observe and learn.

MLM:Kanyoo oshi engipato ino talapa? What is your monthly earning?

RESP: Ore intumorot ainei eabori talapa naa ilakin are. My lowest monthly income is 200,000Tshs.

Demographic information removed

TBS 4 - Arusha

Interview conducted in Maa. Translated/transcribed by ML. Blue text added by EBC listening to the vocal English translations in the original recording

MLM: We are interviewing you because we understand that the knowledge you have is important for the born setting in kilimanjaro region.

RESP: True

MLM: Do you have anything to tell us before we ask you anything?

RESP: No I don't have

MLM: What are your first thoughts when approached by the researchers?

RESP: About the things you are asking me?

MLM: Yes

RESP: I just want to tell you that you are welcome and anything that you want from me I will be ready to answer.

MLM: As a born setter what services do you provide?

RESP: if that is the case, the first thing is that i am not educated therefore i have not gone through school and no one as taught me this, only ability from god's giving. Secondly all patients that went through my treatment has fully recovered and even those who firstly taken to mount Meru hospital fail in treatment and they were brought back to me and they all recover after my treatment and I can say it is only God giving that enable me to perform all this because I am not a learned person therefore i did not learned. The things that I usually used to set a bone of a broken person are brought to me, i don't have them at home, they will have to and bring them for me, I don't have a piece of cloth to wrap on the broken part and i don't have a piece of wood they just go and bring it to me. I don't have a well equipment for this treatment at home as I can not keep predicting people to be broken, i just wait for the patient to be brought to me and that is when i order the equipments after diagnosing the problem, they bring me a bandage, white piece of cloth and a piece of wood. If the broken part in the arm is here.. (showing the arm), I just pull it, stretching it and while stretching the other person is pooling the person at his/her back, I keep on stretching until I see that the bone has return to its initial space and then i tied it and tie the arm on the neck with a rope and keep visiting to observe the progress. I sometime spend two weeks or three weeks and check to see if the patient has recovered. After two months I untie the patient and allow him/her to go. So generally I don't have materials at home to practice this treatment.

MLM: Apart from the bone setting service, which other service do you provide to the people?

RESP: The second service that I provide is helping cows during birth failure. When the calf inside the cow is having some mislocation that make birthing difficult, then I can enter the hand into the animal and correct the mislocation and proceeding in removing the calf alive by pooling it using a rope that insert on the neck.

MLM: Do the rope survocates the calve?

RESP: No it doesn't survocate because I will be controlling the rope.

RESP: If i found a cow with a bis ceas in the womb that make birthing difficult I can operate it and remove the calf safely. If a find a person with the pupil inflammation or swelling then I torn the pupil using a needle and remove the eye content, then I put a little amount of soda ash, a sheep is slaughtered and the patient is given sheep oil and when the person recover the shape of the eye remains although a big number of them lose their vision but both eyes will be having the same shape despite one losing its seeing ability. I have already treated four people and there is one who has gain vision in the defected eye I think because i treated her at a younger age. Only God has shown me this as i have never gone to school. Also when the uterus of cow came out I wash it with warm water and soap and return back into the animal and the animal continue being fertile.

[TBS4.2 0:10] MLM: Did you tell us all the services you provide or there is more?

RESP: The only thing to add is that I have just treated more than two hundred people although I have no clear written records. People are no longer taking people to hospital all this neighbouring, they just bring them to me.

MLM: Is this work the only source of income?

RESP: I have no other work only this one.

MLM: Do you keep animals?

RESP: Yes i am a pastoralist

MLM: How long have you been treating people as bone setter?

RESP: It is a long long time.

MLM: Is it when you are a moran?

RESP: I was not a moran I was just a junior elder in 1987 when the younger Ilkorianga are being born.

MLM: How did you started treating broken people?

RESP: I started setting bones in broken animals, after that I started driving that idea to people after I so that the animals are recovering. People brought to me the first broken person assuming that since I set animals bones than i might be able to set people as well. After I tried that one he recovered and the trend continues and I have already set more than two hundred people.

MLM: Who showed you the skill of setting animals bones?

RESP: It is just God who showed me this, I just started setting animals as I am a livestock keeper.

MLM: When did you started setting broken animals?

RESP: I started when I was a moran, more than thirty years ago.

MLM: You told us it's God who showed you all this, can you tell us how God showed you this?

RESP: I don't know how but it is God who gave me courageous in trying things.

[TBS4.2 8:34] MLM: Where did you treated your first patient?

RESP: In this area.

MLM: Do you set peoples bone the way you do to animals?

RESP: Yes I do the same.

MLM: Are there changes as you practice bone setting?

RESP: Yes there are changes, in the beginning I don't use a very long piece of wood, but as time goes I discovered that if use a long piece of wood the patient recovered quickly because it restrict movement.

MLM: Is there any other bone setter in this neighbourhood.

RESP: No

MLM: Have you ever received any training about this training?

RESP: I have never received any training. I also have my personal assistant and he has started knowing how to perform this treatment as he is the only person who is courageous.

MLM: Is he your brother? How do you related to each other?

RESP: Yes he is my brother from another mother.

MLM: Do you usually train him?

RESP: I don't teach him, he has just observed me doing it until he became an expert and he has already treated four people and they were successful.

MLM:Can you please tell us how you set the bones of a broken person?

[TBS4.2 15:42] RESP: After the patient is brought to me, I diagnose to find the problem. When i discover a dislocation or broken bone, then I massage it using oil/gel bought in the shops until the bones came to its position. Then after the bones has come to its position I tied the arm or leg with piece of soft cloth so as to prevent any scratches from the wood that is used to tie the broken part. Also putting a soft cloth or wrapping the pieces of wood with a soft piece of cloth before tying will ensure quick recovery. After tying the arm with this pieces of wood, i tied the arm firmly to the neck to restrict movement and ensure that the arm recovered quickly.

MLM: Where do get a piece of cloth and the piece of wood to tie the broken arm?

RESP: I just make piece of cloth by tearing a heavy maasai shuka and use it to tie the arm. And if the person has a broken clavicle, I use a black big sellotape and make piece wood equal to the size of a clavicle, then I press the clavicle bone using the piece of wood and then tie using the sellotape. After that the person shoulders are tied using a piece of cloth and the person recovered.

MLM: How large is that piece of wood?

RESP: The size of the wood is just the size of two fingers put together. I had a case of a person involved in an accident. Two people died and one was thrown by a car hit on a stone and the hip bone was broken in its lower part. I lay the person on my thighs and press the shoulders and the legs until i discovered that there was a mislocation in the hip bone. (Check for the hip instability test) I massage the

patient and tied the broken bone with piece of wood and the person has recovered and went back to his boma.

MLM: Why do you lay the person on your thighs?

RESP: In order to see the broken place as i will have to press the legs and the shoulders

MLM: Where do you get the piece of woods?

RESP: They just bring me some left overs pieces of woods in the building sites and I smoothen it myself..

MLM: Do you use anesthetic?

RESP: I don't have anesthetic at all, the person will have to tolerate but after treatment I gave them some herbs.

MLM: How long do you massage the patient?

[TBS4.2 34:40] RESP: It doesn't take long time, three to five minutes.

MLM: What is the name of the herb you give the patient after treatment?

RESP: The patient is given iloodwa and olmisigiyioi

MLM: How do you prepare those herbs?

RESP: When i finish treating a patient they bring iloodwa and grind them, mixing it with olmisigiyioi, then cooked until the mixture is red in colour, they make porridge using maize flour and it is given to the patient. And if the sheep is slaughtered for the patient, then he/she uses the herb as drinking water and the patient is prohibited from taking milk

MLM: Which parts of this herb do you usually use?

RESP: For olmisigiyioi i use the stem and for iloodwa i use the fruits.

MLM: Do you always change the piece of cloth and the piece of wood that you used to tie the broken part or you just leave them until the patient recover?

[TBS4.2 37:54] RESP: Wait after 2 weeks, I just keep checking so that when the broken part reduce in swelling then I re-tie again

MLM: Do you re-tie with same same materials or new one?

RESP: The piece of clothes are washed dried and then i re-tie the patient broken part with the same.

MLM: How long does the patient take to recover?

[TBS4.2 38:48] RESP: The patient might take up two months to recover. Longest is 2 months, shortest is 1 month

MLM: Do you allow the patient to move while stilll sick?

RESP: The patient is taken back to his home after treatment, then i keep visiting him, they take me with a bodaboda. The patient with a broken leg is not allowed to walk until recover but for a patient with broken arm is allowed to walk.

MLM: How many times do you visit your patient?

RESP: Every after two weeks. For arm broken patient and in order to prove whether the patient have recovered, i just take three liters of water and allow the patient to lift and ask if he/she feel pain, then five, then ten if he/she do not fill any pain then I release him/her for he has recovered. For a leg broken patient, I test him by telling him to walk a certain distances if he can not complain pain then he recovered. Before allowing the patient to walk I remove the long wood that was used to tie.

MLM: Do you give the patients any medication after he/she has recovered?

RESP: No i do not make any followup any more.

MLM: How do you treat wounds that result from the bone breaking the skin?

RESP: I had some cases of this kind, i just enter the finger into the wound to remove small bones which might have been left into the wound. After that i massage the protruding bone until it gets in its position. After the bone has entered its position i stitch the wound but leaving gaps to ensure that there is a place to put the herbs through. Even if i will have to tie the place i must skip the wound. If the wound is small, then i will have to enlarge it myself with a knife so that i can get out the small pieces of bones.

MLM: Do you have gloves?

RESP: I don't have i just do bare handed and after that i washed hands.

MLM: What do you use to stitch the wounded part.

RESP: I used a needle we use to stitch bush knife pods and the string that is used to make clothes.

MLM: Do you have the string with you or you buy it in shops during treatment.

RESP: When the patient is brought and need to be stitched, then i send people to buy for me in the shop.

MLM: Do you apply the hospital medicine by yourself?

RESP: The just go and bring them to me i apply them and show the relative of the patient on how to apply the medicine.

MLM: What do use to apply the medicine on the wounds?

RESP: I just pour over the wound using the bottle lid.

MLM: How many times is the medicine administered or applied?

RESP: Four to six times but the interval also increases as the patient recovered. Washes the wound with clean water mixed with salt first.

MLM: Do you leave the wound untied when you tie the broken bones?

RESP: Yes i dont tie the wounded part. The wood does not cover the wound.

MLM: What do you do to people with broken cartilages?

RESP: There was a case of a patient with a broken knee cartilage and stayed for three years untreated. When i found the patient i ordered a goat to be slaughtered during day time after grazing for some time. The after slaughtering I quickly took the stomach of that goat while still hot and the patient entered his leg into the content of the goat stomach. The cartilage is soften by the temperature in the stomach content, then after that I massage the knee with smooth stick until the cartilage stretches to its normal position then I tied it. That patient has fully recover and walked properly.

MLM: How did you know that keeping a broken cartilage inside a stomach content for sometime make it tender?

RESP: It is common among us that when you are injured by a thorn and remain in your flesh and difficult to remove it, we slaughter a goat or sheep and the injured part is put on the stomach content for sometime and then will protrude out after one day. We also know that doing this make the cartilages tender.

MLM: How do you treat people with dislocated joints or back pain or knee joints problem?

[TBS4.2 1:06:17] RESP: Does not treat, I only give the person an advice that he should slaughter a sheep a fat one. Rost the fat about four litters then grind one kilogram of iloodwa then mix with the oil while still hot. The mixture is used by putting two to three spoons of it in foods or porridge. After using the oil for sometime the patient recover his/her back pain or knee joints pain.

MLM: How does iloodwa help the person?

RESP: Purifies all systems in the body's systems, prevents infection. It cleans the blood and improve immunity.

MLM: What does the iloodwa and olmisigiyioi help in a broken person?

RESP: It dries up the wound and prevent swelling. Also increase immunity and avoid other side infections.

MLM: Do you treat long time injuries that make people lame although there is no pain?

TBS4.2 [1:15:30] RESP: Yes i do treat. There are several cases of people taken to mount Meru and there had a POP on their broken legs and they recover but the leg is no longer straight. The problem with mount meru hospital is that they do not stretch and massage the patient leg. The patient might have an overlapping bone due to the accident. In this case the patient need to be massaged to make the bones location correct. Two patient of this case were brought to me one with three years and one with seven years I broke them. The one with three years had a lamed leg, i broke it by creating a fulcrum on a long bone with a stone and using a hammer and reset again he recovered fully and walked like the other normal person. The other person was a lady who remain with a lamed arm for seven years after a POP was done. The relatives of the lady lie that the arm has just stay for two years, then I order her to be brought to me and ask them to bring the hospital doctor to administer anesthetic because the injury was very old so it would be very painful to rebreak. after that, i broke the arm and reset again the lady recovered fully.

MLM: Do you corect bowed legs in infants?

RESP: I have never tried.

MLM: Do you always cut or make a hole on the broken part so that you put the medicine on the broken part?

RESP: No.

MLM: Have you ever cut any of your patient's arm or leg thinking that he/she will never recovered?

[TBS4.2 1:24:01] RESP: I will never do that. I had one case of a Chagga patient where by the hospital decided that his arm must be cut. He reach me through a friend and i treated him and instructed him to keep his treated arm between two buckets of water to avoid mobility. After three months the person recovered fully.

MLM: Are you a spiritual healer?

RESP: No I am not a spiritual healer. (at this point the crowd laughed) I am not a religious man

MLM: How many patient did you treated for a year?

RESP: Very many just in this days four people are under observation and a week ago six people are released. So just in a month about ten patients.

[TBS4.2 1:26:30] MLM: How much do you charged per patient?

RESP: One cow for an adult and one sheep/goat for a children.

MLM: Do people sometime escape from paying after they recovered?

RESP: No at all just sometimes delay in paying. There was one patient whom i treated and forbid him from taking alcohol but he ignored and did not recovered. He was taken to the hospital while the leg is still tied and the hospital just provided medicine as they said i did it correctly. The same person escaped from the hospital without paying bills and and the hospital followed him he paid a bill of one million Tshs. After sometime he had an accident the same place is broken again. His relatives came to me requesting to pay the first bill because it was my right. I treated again the man and he recovered.

MLM: Which treatment do you recommend is it the traditional or the hospital one?

RESP: Did i ever tried any?

MLM: I don’t think so. But which one do you think is the best?

RESP: I don't really know, but there are many patients that were brought back to me from the hospital but i have never referred any to the hospital.

[TBS4.2 1:34:50] MLM: Which hospital equipments do you usually use?

RESP: I use a bandage, iodine ~~and plasters~~

MLM: Is there any case that you can not attempt?

RESP: I don’t have a case that i can not attempt.

MLM: I have ever referred the patient to the hospital or to another specialist like you?

RESP: I have never referred although I receive a number patient from hospital

MLM: Are there any other specialist in this area or your neighbouring?

[TBS4.2 1:37:11] RESP: I am the only one and my helper here who is courageous.

MLM: What if a patient is brought and you can't treat, will you refer him to the hospital?

RESP: May be a patient with other health problems , but for those with bones problem i can do it, even a person with a broken backbone i can still treat.

MLM: Will you agree if you are trained on the hospital procedures?

RESP: Yes I agree.

MLM: Do you have challenges that are hindering your ability in working efficiently?

RESP: Yes i am lacking those hospital equipments, such as gloves or diagnostics.

MLM: Is there anything we did not ask which you think we could ask?

RESP: No at all

MLM: Do you have any question?

RESP: No

MLM: Will you train any of your child about this treatment?

RESP: I will not teach any body except any one who need to observe and learn.

MLM: What is your monthly earning?

RESP: My lowest monthly income is 200,000Tshs.

Demographic information removed

TBS 5: Manyara

Interview conducted in English and Swahili. Translated/transcribed by OS.

EBC; We are interviewing you because we consider your knowledge to be valuable in understanding the practice of traditional bone setters in northern Tanzania.

HM translating; So tunakufanyia haya mahojiano kwa sababu tunaamini utaalam wako na umuhimu wa utaalam wako katika kutibu magonjwa ya mifupa katika kanda ya kaskazini(We are doing this interview because we believe in your talent and the importance of your talent in treating bone diseases in the northern zone)

EBC; Do you have any question before we start

Tbs. sina swali (I don't have a question)

HM. he doesn't have any question

EBC;what was your first thought when you was first approached by our research team

TBS I was very happy when I was approached by the team, I was happy to share my challenges and success stories, and i wish i could have someone to collaborate with i could do a very good job because I am doing a job which is a talent and it's not that I imitated from anyone, I started doing this when I was 19 years old, as you can see in the picture me my mom and my dad treating a child who had a dislocated shoulder, and I am very happy and comfortable with the team here.

EBC what other medical issues do you treat

Tbs I only two diseases fracture and stroke

EBC oookay do you treat both open fracture and closed fractures?

Tbs I treat both open and closed fractures

EBC does he also treat dislocations

Tbs I can also treat dislocations

EBC do you treat ligament and/or tendon injuries

Tbs ligament and tendons? No

EBC and aaam chronic bone injuries like osteoarthritis and something like that

Tbs yes

EBC; ok and two more so old fractures that have already healed

Tbs so for malunion I collaborate with anaesthetist or a doctor, a real doctor, we give the patient anaesthesia then we do closed osteoclasis and rearrange the bone and then the patient gets well

EBC great…

HM we should clap hands for him on that clap….clap…clap….very impressed

EBC and lastly do you also treat congenital malformations ??

Tbs they are correctable, I do treat them, I have helped many, they are born with deformed limbs and unable to walk normally when they come I do treat them but they have to come early when the child is still young

EBC okay ….and so what parts of the body do you treats…

Tbs everything that has to do with bone,

EBC okay everything

Tbs yes that has to do with fractures and stroke and ligamentum injuries

EBC so okay…sounds good

EBC so you mentioned that your father taught you your skills, how long did you learn with him and how did he teach you

Tbs ..At home …Musoma

Tbs . He told me I have to pull the limb with steady force until I get a satisfactory reduction then take pieces of wood which put them on the injured limb circumferentially , hold them with a gauze then open weekly to check alignment ..if the alignment has been lost re do the reduction…it takes time to heal.

HM how many weeks

Tbs for the Ieg can say its one month

HM he/she will be healed?

Tbs yes

HM how about the femur?

Tbs for the femur it takes two to three months

HM how about the upper limb ??

Tbs for the upper limb it heals faster but people are different as there are people whose bones heals faster while others delay.

HM how do you know that the bone of this particular patient will heal faster?

Tbs I know that after starting treating the patient.. because for a example there are people I gues because I have not studied it maye be its because ofdiet and vitamins that are causing the bone to delay uniting .

HM its true

HM so we have seen the materials , there are pieces of wood and aaaah hospital bandageslike these ones

EBC okay

HM and…even cotton wool to wrap around before …to wrap around before aaammmm….

EBC yeah…

HM don’t move don’t shake…

EBC yees ….continue…

HM aaam so the materials as like you saw those pieces of wood aaam,hospital bandages and hospital cotton, to wrap around the leg,first of all he pulls the leg arranges the bones to bring the bone into alignment

EBC mmmm

HM reduces the fracture in other words and then ammmm and from there he will wrap the pieces of wood around the broken bone, to maintain the reduction and every other week he will be checking the patient

EBC okay

HM to see if the alignment is still the same and still maintained

EBC okay

HM from thereee..for the tibia it takes like a month for the patient to heal

EBC mmmh

HM for a femur like two months, radius and ulna less than a month.

EBC okay… and when he is reducing the fracture he pulls the leg ,does he also do massage

HM yes

HM and how does you find where the fracture is…

Tbs some I just palpates the fracture some I uses an x ray film

EBC okay mmmmh and where do you get the x ray

tbs so I do sends the patient to take an x ray somewhere in town

EBC okay…so the patient goes to get that x ray in moshi for him….and where do you get the wood and the bandages

Tbs I do buy a big piece of wood then I chops it into tiny pieces of the size that I wants.

EBC okay

Tbs … the other gauze bandages ,,I gets them from a pharmacy

EBC okay…mmmh

HM yes

EBC and then after the wound has healed…the fracture is splinted is there any period of immobilization or any kind of exercise that happens , and when does that starts

Tbs yeah so I gives them a grace period aaa sometimes before they start walking on that broken limb

EBC okay

EBC okay do you give the patient any anaesthetic ..analgesia?

HM yes he sends patient to the pharmacy tell the pharmacist to give you this pain killer.

EBC aaa ok

EBC okay and next question

OS the most common ?

Tbs one doctor told me tramadol is good ,,,so I do use tramadol very often

EBC okay …and do the patient recover here or do they go home

Tbs so here the space is tiny I don't have much space and much more rooms but back home my dad used to have so many houses that he had even a ward a patient will come in with the relative , the relative and the patient will have to stay in until they are healed.

EBC where is the ward

Tbs ...i mean back home where my dad was treating patients

EBC aaa okay okay okay

EBC waaoh….okay and so after the fracture has healed is there any follow up clinic or do he give any medications to the patient? After they healed ,,,after the fracture has healed

Tbs I do give them a follow up and aaa until the patient is can run or if it's a hand fracture ,he gives you a machete and then asks you to cut a piece of a tree if the patient don't feel pain I discharge him/her completely from the clinic.

EBC okay

OS so on average how many clinic visits a patient makes before being discharged completely from the clinic ?

Tbs for upper limb fractures its 4 visits and lower limb fractures its 8 visits

EBC, and do you have someone helping you ?

Tbs yes ..my wife is a helping hand, although she a bit scared with nasty fractures

EBC m

EBC okay, so can you take us through the process of treating an open fracture

[TBS5 22:11] Tbs what I do , when apatient comes in with an open fracture i will reduce the fracture by pulling and then there is a nurse near by.. a medical nurse near by I will call that nurse , the nurse will do dressing using antiseptic and all other hospital medications and then I will also prescribe some antibiotics to clear the infection and I will be opening the wound every other after three days for wound dressing

EBC okay…so the wound dressing is changed every three days

Tbs yes ..every three days

OS so here there are more follow up for open fractures than closed fractures

Tbs , yes for open fractures the follow ups is every three days

EBC, how do you decide which kind of antibiotics to buy ?

Tbs I just send the patient to the hospital and the medical practitioner will decide which drug to give.

HM , do you think the medical practitioner will change the management plan?

Tbs no , most of the patient s will not agree. Patients allow the doc to take care of the wound only, the fracture is for me. there was one doctor who was my friend but now he is not around I used to call him and ask him to take care of the wound.

EBC why the patient not agreeing to different treatment at a hospital

Tbs because they trust me ..

EBC can you take us through a process of treating an old fracture that has healed in malunion?

Tbs if a patient comes with a malunion I wil rebreak the fracture and re align it and for those who are sensitive to pain they to pay ten thousands to call a doctor who will bring anaesthetic drugs and anaesthetize the patient

EBC ok, local anaesthesia or general anaesthesia ?

Tbs local anaesthesia

EBC and how do you break the bone …how do you re break the bone

Tbs there is a drug called ubende in jaaluo language which I apply on the fracture site and then use a hot machete to massage the fracture site and make it easy breakable…..its made from different leaves and they are only found in musoma and when its out of stock ,he has to find mony and travel to musoma for refilling …he can not mention all the leaves because the names are hard to pronuouce …..????secret recipe ??

EBC, can you take us through a process of treating a congenital malformation??

Tbs a child with congenital malformation I use this drug, and there is this drug which I use it in patients with stroke, I will just sponge the patient until he is healed and I do the same for crooked limbs. and the earlier you do it the better.

EBC, do they get any splinting after the malformation is corrected?

Tbs some need splinting ..some don't …

EBC can you take us through a process of treating an old injury such as osteoarthritis ?

Tbs , this is the herb I use, if you have back pain I lay you there and massage your back, this is a recipe from musoma

HM , he is saying he can not say the name , I think this is because he is afraid people will copy his ideas and use it to treat …..secret recipe

EBC, can you take us through a process of dislocation?

tbs, I relocate it manually and splint it with a bandage in this position

[TBS5 39:18] EBC moving back into open fractures .. what do you do if the limb dies?

tbs , there are signs like the toes changing into black colour is a sign that the limb is not viable so when I see them I will refer the patient right away.

EBC , where do you refer the patient to ?

Tbs , I do refer them to NKOARANGA AND KCMC

EBC do you use any scarification technique

Tbs no.

HM, I was once treated with scarification when I contacted chicken pox , and my grand ma took me to her friend who treated me with scarification

EBC, do you provide any kind of spiritual treatment?

Tbs I do not provide spiritual healing but most of the time I do pray before starting treating a patient… and am a protestant

EBC, how many patient do you treat per year ?...by approximation

Tbs for those who I treat and they don't have money I do write them in this book, per week I treat 30 patients(new and follow up cases), and I new cases ranges from 3 to 10. The average is six …so its 312 new cases per year

EBC so do these patients stay in a ward

HM, no he doesn't have award, his father had a ward .

EBC so where do this patients stay till recovery

HM , they go home .

EBC how do the patients finds out about your practice

Tbs its from word of mouth and its from those who have been treated here, they go and spread the good word

EBC, how much do you charge per treatment?

Tbs , it depends on the patients financial status , the maximum I have received is 500k and for those who are poor I sometimes don't charge them

EBC how do you determine how much the patient should pay

Tbs is after assessing the fracture he will give you the price and if you cant pay all the money he will write your name in this book and you will bring the money when you get .

HM, how about follow up clinics ?

Tbs, for follow up clinics its free. once you pay on the first visit it covers the follow up visits .

EBC, what do you think of the care of bones provided by hospitals .

Tbs some people in hospitals they apply full cast pop on the first day and schedule the patient for a follow up clinic after six weeks when the swelling subsides the pop become loose it does not works well, this is my concern about hospital treatment, but otherwise am okay with hospital treatment .

EBC, we know you use x rays and you call a nurse for antiseptic technique do you ever used calcium plaster pop??

Tbs no

EBC do you ever gave iv medications

Tbs no

EBC do you use disposable gloves ,

Tbs , yes , here they are ..

EBC, do you perfom any procedure such as suturing

Tbs no

EBC, do you feel there are cases you can not treat ?

Tbs , yes, like there was a kid who was brought here with necrotic gangrenous limb , so he advised the parents to take the child to a hospital , and they did that and later on he called them to ask them what happen , and they said the limb was amputated.

EBC have you ever referred a patient to another traditional bone setter?

Tbs , no

EBC, do you feel there should be improvement in care of broken bone whether in hospital or tbs

Tbs very much needed , so many people who are poor are sustaining bad injuries and you can’t help them because they can’t afford to cover the bils

EBC so okay,, what kind of change would you make if you could?

HM, suppose you was president magufuli, what will you do to improve the situation?

Tbs , if I had to change anything I will improve the quality of the services and the environment that this tbs are working on like to attach a tbs with maybe a nurse or a doctor who will be giving pain medications before procedures, to build a ward where patients will stay until they recover , he gave like three examples of patients he treated like last week, a kid who had fracture and no one to support , was

brought here and he helped, and another kid who was brought here with her mom and they had only 5k, he helped because he is also a father and for him money is nothing but the humanity in him, he has also kids.

EBC, will you be willing to learn how to perform hospital technique to treat??

Tbs, yes am willing.

EBC, is there anything else you want to say

Tbs , I like to be trained but am worried how I will leave the family here, I can not leave the family starving and go sit in a class.

EBC, is there anything that you think we should have asked but we didn't ask ?

Tbs , no, you have exhausted all the information,

Demographic information removed

TBS 6: Manyara

Interview conducted in English and Swahili. Translated/transcribed by OS

HM, Sir the importance of us coming here is first we recognize your contribution in treating bone diseases in northern Tanzania and you have being helping people who in one way or another they can not reach to hospitals , we value what you are doing and that's y we came her to have this interview with you., so before we start do you have any question ??

Tbs , The first question is if this interview is for a good reason I will explain to you what am doing comprehensively because I have been treating people very nice

HM , sir this interview is very good and even you will realize how good it is after hearing this questions

HM, what types of patients do you treat

Tbs , I treat patients with fractures of the all bones both open and closed

HM, do you also treat dislocations and tendon and ligamentum injurues ??-

Tbs , also ligaments and tendons and clavicles

HM, where did you learn this

Tbs , from my father

HM how long did you learn this

Tbs ever since i was young my father used to call me as a helper while he was doing fracture fixation to todate

HM, he has not received any formal training on bone treatment

HM, did you go to school?

Tbs no

HM, is bone setting your full time job

Tbs , no I am also a pastoralist

EBC, does he treat anything else other than bones?

Tbs only bones

HM, what materials do you use when treating bones ?

[TBS6.1 7:44] Tbs gloves, gauzes, for open fractures and splitting with dried cows skin, he obtain them from pharmacy and the cows skin he get it after they slaughter a cow

HM, so for example when someone sustain a forearm fracture, how long does it take to heal

Tbs in children its less than a month, for adults it's up to six months

HM, do you see your patients regularly?

Tbs , yes , they come here and in acute phases I accommodate them in my compound until they are stable

HM after how long do they come to clinic after being discharged

Tbs , whenever the splints become loose the patient will come and I will re apply it, and for other patients I do visit them at their houses while other will come by themselves

EBC how often

Tbs until it gets loose

HM sir do you give local herbs for pain and healing?

[TBS6.1 19:33] Tbs , yes , it's a root and called emsigie, its oral medication

HM how do you measure the dose, one cup per dose, PRN

EBC how do they prepare

Tbs by boiling the roots then drink the soup from the roots

HM sir do you do anything to prevent infection?

Tbs , I instruct patients with open fractures to go to hospital to get tetanus injections

HM do you give local herbs to prevent infection?

Tbs , I do give the goat oil to drink and it speeds wound healing

HM do you close the wound ?

Tbs , no , I do left it open

Tbs , son …when a patients comes with an open fracture , he sends somebody to the pharmacy to buy antiseptics and he will wash the wound thoroughly with antiseptic.

HM, what medication do you give when you splint a limb and you find there is colour change of the fingers after sometimes

Tbs , only oil I give him to drink and a herb and they do recover well

HM, whats the name of the herb ,

Tbs , its called ndungwi, it's a branch which I get it from the bushes around here and I burn it to ashes then apply the ashes on the wound

HM, do you do scarification ?

Tbs no

HM do you provide spiritual treatment ?

Tbs no

HM how many patients do you think you treat per week? Month? Year??

Tbs son….. up to 10 new patients per week …average of 500 patients per year

HM, how does the patients finds out about mzee?

Tbs son , its from word of mouth by the patients which has been treated by mzee , they testify to other patients

EBC how do they recover

Tbs son ….its depends with the age of the patients , in children its faster than adult, and also the environment and the type of fracture will affect rate of healing

HM how do you know where the fracture is…..

Tbs I look

Tbs son , he feel it with his hands and locate the fracture

HM, how much do I have to pay for service when I come with a fracture …in average

Tbs , for children is a goat and for adults it's a calf …cows calf …..i don't prefer cash

HM . what if I bring corn ?

Tbs , no I don't take corn …

HM .. what do I have to give if I don't have a goat or calf

Tbs 100k to 300k and if you wish you can give more dependending on how you are satisfied with the care you received

HM, what's your say about the care hospitals provide, if you fall sick will you go to hospital ?

Tbs I will go and I don't have problem with hospital care

HM what kinds of hospital equipments do you use

Tbs gauzes , gauze bandages , antiseptics and tetanus

HM, are there bone diseases which you think you can not treat them ?

Tbs ,yes …like those severly comminuted

HM, have you ever referred patient to another tbs ?

Tbs no , when I cannot treat a patient I do send him/her to hospital preferably kcmc/moshi arusha

HM…..are you willing to learn hospital technique ? like if the government is willing to train you on treatment of fracture, if if will cover everything

Tbs , it's a good thing

HM, what do you think we can do to improve your services

Tbs , if I get someone to anesthetize the patient it will be a very good step because now what we do is the patient is held with strong men to prevent him being uncooperative during the procedure because of pain. And how to apply a pop instead of this goat skin ?

HM , what do you do when a patient comes with a malunion?

Tbs I re break the bone

HM ho do you rebreak the bone

Tbs , we apply a fresh intestine of a goat around the fracture site and the fracture site will become easly breakable because of the hotness , if you don't do this you may create another fracture

HM do you remove the contents ?

Tbs , we leave it , that's the drug

HM , for how long do you keep the intestine

Tbs son….20 minutes

HM, you learned from your father , did he also teach one of your sibling ?

Tbs , yes there is one , but he's very old to do it now

HM , are you planning to pass this to one of your children ??

Tbs son … there is one who has started treating bones and fractures but as of now he's good with fractures of the upper limbs and he is still learning about the lower limbs and intra articular fractures …..he is still learning .

HM , is there anything else you want to say ??

Tbs , its about training me and helping me getting a ward to keep my patients

HM whats the maximum number of admissions you have ever admitted

Tbs son …..3 to 4 patients

HM ,,,, do you train people from other family

Tbs no

HM … is there anything that you think we should have asked but we didn't ask ?? or you expected us to say but we didn't say ?

Tbs …you asked and said it all but its really giving me headache how to accommodate my patients as I have said I have a limited space

Demographic information removed
